# The connection between the dynamic remodeling of the mitochondrial network and the regulation of muscle mass

**DOI:** 10.1007/s00018-020-03662-0

**Published:** 2020-10-19

**Authors:** Vanina Romanello, Marco Sandri

**Affiliations:** 1 Venetian Institute of Molecular Medicine, via Orus 2, 35129 Padova, Italy; 2 Department of Biomedical Science, University of Padova, via G. Colombo 3, 35100 Padova, Italy; 3 Department of Medicine, McGill University, Montreal, Canada

**Keywords:** Atrophy, Mitochondria, Fission, Fusion, Mitochondrial proteostasis, Autophagy, Mitophagy, Skeletal muscle, FGF21, Myokines

## Abstract

The dynamic coordination of processes controlling the quality of the mitochondrial network is crucial to maintain the function of mitochondria in skeletal muscle. Changes of mitochondrial proteolytic system, dynamics (fusion/fission), and mitophagy induce pathways that affect muscle mass and performance. When muscle mass is lost, the risk of disease onset and premature death is dramatically increased. For instance, poor quality of muscles correlates with the onset progression of several age-related disorders such as diabetes, obesity, cancer, and aging sarcopenia. To date, there are no drug therapies to reverse muscle loss, and exercise remains the best approach to improve mitochondrial health and to slow atrophy in several diseases. This review will describe the principal mechanisms that control mitochondrial quality and the pathways that link mitochondrial dysfunction to muscle mass regulation.

## Introduction

Mitochondrial dysfunction has been linked to muscle function loss that occurs in several age-related metabolic disorders such as diabetes, obesity, cancer, and aging sarcopenia. In these conditions, the decrease in muscle mass is a significant health problem that worsens life quality and increases morbidity and mortality. Instead, maintaining a healthy skeletal muscle mass is associated with a lower risk of mortality [1, 2], highlighting a correlation between muscle health and whole-body homeostasis. Skeletal muscle, the most abundant tissue in the human body, is a major site of metabolic activity that regulates carbohydrates, lipids, and protein homeostasis. Energy requirements during intense contraction in skeletal muscles increase to 100-fold the consumption of ATP [3]. To sustain this high energy demand, cardiac and skeletal muscles rely on oxidative phosphorylation (OXPHOS) for ATP production. Therefore, maintaining a functional mitochondrial network in this tissue is fundamental to sustain the metabolic demands imposed by contraction, ultimately regulating fuel utilization, energy expenditure, and general metabolism.

## Signaling pathways regulating muscle mass

The muscle mass in adulthood is defined by the dynamic balance between protein synthesis and protein degradation. Mechanical overload or anabolic hormonal stimulation shifts the balance toward protein synthesis with consequent increases in fiber size, called hypertrophy. Conversely, in catabolic conditions, protein degradation exceeds protein synthesis leading to muscle weakness and muscle atrophy. The main pathways controlling muscle size are IGF1–AKT–mTOR–FoxO and TGFβ/myostatin/BMP signaling. The Smads transcription factors regulate muscle mass downstream of the TGFβ/myostatin superfamily of ligands. Myostatin-dependent recruitment of SMAD 2/3 is a negative regulator of muscle mass [3]. Accordingly, inhibition of SMAD 2 and SMAD 3 is sufficient to induce muscle growth [4, 5]. On the other hand, BMP signaling is a positive regulator of muscle mass and is dominant over the myostatin pathway [6, 7]. BMP pathway is activated in myostatin knockout and is sufficient, when induced, to promote muscle growth and to counteract muscle loss after denervation and in the absence of nutrients [6–8]. Induction of BMP signaling activates Smad1/5/8, which, together with SMAD 4, leads to the suppression of the MUSA1 [6] and the activation of mTOR-dependent protein synthesis [7]. The IGF1–AKT-–mTOR axis increases protein synthesis by stimulating the translational machinery and simultaneously blocks FoxOs transcription factors and protein degradation [9]. Two central ATP-dependent proteolytic systems are activated during muscle atrophy. The ubiquitin–proteasome system degrades predominantly myofibrillar proteins, whereas the autophagy–lysosome system removes dysfunctional organelles, protein aggregates, and unfolded and toxic proteins. Muscle atrophy requires the activation of gene transcription programs that regulate the expression of a subset of genes that are named atrophy-related genes or atrogenes [10–14]. These atrogenes belong to several fundamental biological processes such as the ubiquitin–proteasome and autophagy–lysosome systems, protein synthesis, ROS detoxification, DNA repair, unfolding protein response (UPR), mitochondria function, and energy metabolism. FoxO family of transcription factors (FoxO1, FoxO3, and FoxO4) are critical mediators of the catabolic response during atrophy [9, 15, 16]. Muscle-specific inhibition of FoxOs protects from cancer cachexia-, fasting-, hindlimb suspension- or denervation-induced atrophy [16–18]. Moreover, at least half of the atrogenes require FoxOs for their up- or downregulation [16]. FoxOs- dependent atrogenes include the E3 Ubiquitin ligases ATROGIN-1, MuRF-1, MUSA1, SMART, several autophagy-related genes such as LC3, GABARAPL1, BNIP3, CATHEPSIN L, and the ER stress genes ATF4, GADD34 and GADD45 [9, 10, 15, 16]. Importantly, the overexpression of ATF4 and GADD45 is sufficient to induce muscle loss [19, 20]. Therefore, by controlling FoxOs and mTOR, AKT is a regulatory node between catabolic and anabolic processes. However, muscle atrophy involves not only the breakdown of the myofibrils but also the loss of organelles like mitochondria. Moreover, mitochondrial function and, thus, energy production are generally reduced during muscle wasting. More than 10% of the atrophy-related genes are directly involved in energy production. Genes encoding for glycolysis and oxidative phosphorylation enzymes are coordinately suppressed in atrophying muscles during denervation, disuse, diabetes, cancer, fasting, and chronic kidney disease [10, 11, 13, 20]. This finding suggests that alterations in the mitochondrial network contribute to muscle atrophy. Recent data show that retrograde signaling from the mitochondrial network to the nucleus adapts muscle function to the physiological or pathological demands.

## Mitochondrial communication is essential for optimal mitochondrial function in skeletal muscle

### Mitochondria populations are connected to support energy distribution in skeletal muscle

Adult myofibers have a specific subcellular distribution of distinctive mitochondria populations that have been classified accordingly to their localization as subsarcolemmal and intermyofibrillar. Subsarcolemmal (SS) mitochondria have a globular shape and are located just beneath the plasma membrane (sarcolemma). A fraction of these surrounds capillaries and nuclei (perivascular and perinuclear mitochondria, respectively) [21]. The intermyofibrillar (IMF) mitochondria are elongated with a tubular shape [21] and are inserted among myofibrils arranged in pairs at the z-line of each sarcomere [22]. While less is known regarding perivascular and perinuclear mitochondrial function, several reports have identified differences in the SS and IMF's biochemical and functional properties [23–25]. Recent findings on high-resolution three-dimensional microscopy challenged the existence of two separated mitochondrial pools and strengthened the concept of a physically and functionally highly interconnected mitochondrial network in skeletal muscle [21, 24, 26]. The mitochondrial muscle reticulum forms a conductive pathway that can rapidly transfer energy from the oxygen source, the capillary, to the contractile apparatus [24]. The physical connection of SS and IMF in human and mouse muscles enables the distribution of the membrane potential from the subsarcolemmal region, where respiration happens to the intermyofibrillar is where the ATP synthase complex uses the proton gradient to generate ATP for myosin–actin interaction.

### Interorganelle communication relies on intermitochondrial junctions and nanotunnels

In addition to SS and IMF connection, mitochondria also communicate among themselves through intermitochondrial junctions (IMJs) and nanotunnels. IMJs are highly specific and regulated electron-dense structures, defined by the close contact of the inner and outer mitochondrial membrane. These structures allow the coordination of cristae orientation and electrical coupling between adjacent mitochondria within the mitochondrial network [27]. To prevent the spread of dysfunction, IMJs are detached in dysfunctional mitochondria, promoting the electrical isolation and further segregation from the network of the malfunctioning organelle [28]. IMJs’ function and molecular composition have to be defined, but one possibility is that they are ion channels explaining the rapid electrochemical inter-mitochondrial communication [27]. Nanotunnels are conserved OMM and IMM double-membrane projections, connecting two non-adjacent mitochondria [29], particularly under pathological conditions [21]. These tubular structures can transport ions, metabolites, and proteins, between spatially restricted mitochondria like the myofibril-embedded IMF [21]. Thus, advances in 3D imaging have expanded our knowledge of the dynamic mitochondrial communication and energy distribution across the muscle reticulum that provides a rapid mechanism to respond immediately to changes in energy requirements.

## The conformation and connectivity of the mitochondrial network are tailored to the metabolism and contractility of each fiber type

Skeletal muscles are composed of specialized fiber types which differ in their mitochondrial content, metabolic properties, and myosin composition. According to the functional demand, skeletal muscles recruit the most suitable myofibers to modulate the expected response. Muscles that produce a long-lasting contraction, like postural muscles, are mainly composed of slow b-oxidative fibers, which have high mitochondrial content, increased reliance on OXPHOS, and are resistant to fatigue. Instead, muscles that generate a high-intensity activity for short periods (e.g., jumping, kicking), have a high representation of fast glycolytic fibers, which have poor mitochondrial content, decreased reliance on OXPHOS and are fatigable. Based on myosin heavy chain (MHC) expression, mouse muscles contain four major fiber types, slow type 1 and fast 2A, 2X and 2B; while human muscles contain three major fiber types, slow type 1 and fast 2A and 2X [30]. The mitochondrial conformation within myofibers has a fiber-type-dependent specific pattern. Oxidative fibers have a grid-like mitochondrial network conformation, with parallel and perpendicularly oriented elongated mitochondria. In contrast, the mitochondrial network in glycolytic fibers is fragmented and perpendicularly oriented to the muscle contraction axis and the I bands. The mitochondrial connectivity within the network through IMJs is higher in oxidative than in glycolytic fibers, reflecting the cell's functional demands. Therefore, the mitochondrial network morphology, arrangement, and connectivity adapt to each fiber type's specific functional needs (e.g., OXPHOS capacity and contractility) [31, 32].

## Quality control pathways finely tune mitochondrial function

**Fig. 1:**
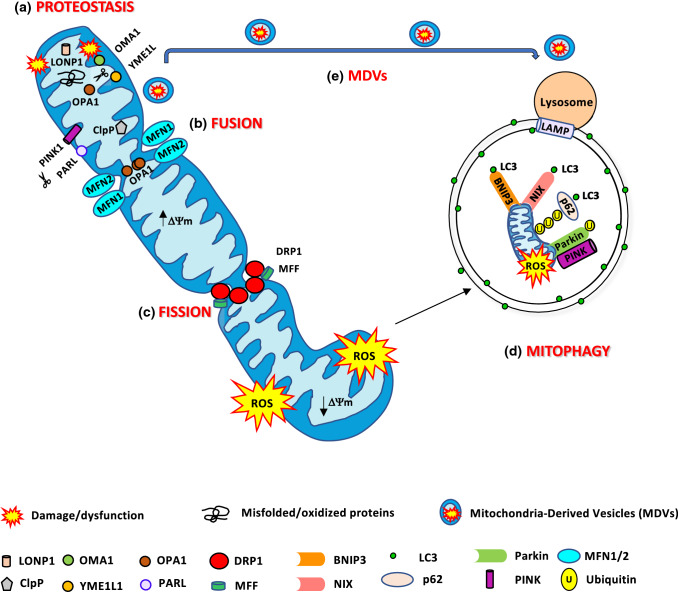
Mitochondria quality control pathways. **a** The mitochondrial proteases LONP1, ClpP, OMA1, YME1L1, and PARL maintained mitochondrial proteostasis. PARL, OMA1, and YME1L1 process OPA1 protein, critical for mitochondrial fusion and cristae remodeling. PARL degrades PINK1, regulating mitophagy. **b** Mitochondrial fusion is mediated by MFN1/2 and OPA1 to produce an elongated mitochondrial network. **c** DRP1 and MFF are the major proteins involved in mitochondrial fission. Fragmented mitochondria with low ΔΨm are removed by mitophagy. **d** BNIP3 and NIX are mitophagy receptors that bind to LC3 to tether mitochondria to the autophagosome. PINK1 accumulates on of depolarized mitochondria surface, where it phosphorylates ubiquitinated OMM proteins and the Parkin UBL domain. Parkin will further promote the ubiquitination of the outer mitochondrial membrane proteins. Then, the ubiquitinated proteins can be recognized by the p62/SQSTM1 adaptor, to initiate mitophagy. **e** Mild mitochondrial damage activates the release of mitochondrial-derived vesicles (MDVs) containing mitochondrial components for their degradation in the lysosome

Mitochondrial dysfunction has been linked to several human diseases, like specific genetic defects, neurodegenerative and age-related diseases like aging sarcopenia, diabetes, and obesity. Mitochondria are continuously challenged by reactive oxygen species (ROS), an inexorable by-product of oxidative phosphorylation. For this reason, the organelle is susceptible to DNA mutations or protein misfolding. Thus, mitochondrial integrity and function need to be highly regulated. Mammalian cells contain several mitochondria quality control systems to preserve the organelle homeostasis. According to the degree of mitochondrial damage, different pathways can be activated, ranging from the segmental repair of the damage to the whole degradation of the dysfunctional organelle. Mitochondrial homeostasis is ensured by the coordination of pathways like mitochondrial biogenesis, mitochondrial dynamics, and degradative pathways like the activation of mitochondrial proteases, mitochondrial-derived vesicles, and mitophagy, the selective degradation of mitochondria via autophagy (Fig. 1).

### Mitochondrial proteostasis—a mitochondrial protein quality control mechanism

Mitochondria have their genome, transcription, and translation machinery [33]. However, only 13 proteins among the approximately 1200 proteins from the mitochondrial proteome, are encoded by mitochondrial DNA (mtDNA) and synthesized inside the organelle [34]. The nuclear genome encodes the remaining proteins, which are synthesized by ribosomes in precursor forms. The incorporation of these precursors into specific mitochondria subcompartments such as the outer mitochondrial membrane (OMM), the intermembrane space (IMS), the inner mitochondrial membrane (IMM) and the mitochondrial matrix requires specific and highly regulated import machinery [35]. The transported proteins are unfolded, with a high risk of misfolding, aggregation, mislocalization, and damage. For this reason, mitochondria contain a complex interconnected quality control system responsible for the maintenance of functional proteins. A recent proteomic study in Drosophila fibroblasts identified that most of the mitochondrial protein turnover (70% *circa*) occurs through the combination of non-autophagic degradative processes like mitochondrial proteases, the ubiquitin–proteasome system, and mitochondrial-derived vesicles; while the contribution of mitophagy to protein turnover is of the remaining one third [36].

#### Mitochondrial proteases

The first line of defense against mild mitochondrial damage involves the degradation of misfolded or oxidized proteins by activating specific mitochondrial proteases in each mitochondrial compartment. In the mitochondrial matrix, protein turnover is controlled by 3 AAA proteases: the soluble LONP1 and ClpP and the membrane-bound m-AAA. Protein degradation in the IMS is controlled by the membrane-bound i-AAA YME1L1, the soluble HtrA2/OMI, the metallopeptidases OMA1 and the rhomboid protease PARL. Mitoproteases do not only monitor mitochondrial protein quality, but also they can decide mitochondrial fate. For example, m-AAA, YME1L1, HtrA2, OMA1, and PARL cleave the profusion protein OPA1 affecting mitochondrial morphology and function [37]. PARL modulates mitophagy by degrading the mitophagy protein PINK1 [38].

#### Ubiquitin–proteasome system (UPS) and mitochondria-associated degradation pathway (MAD)

In mouse cardiac muscle, the UPS ubiquitinates several OMM, IMS, and matrix mitochondrial proteins [39, 40]. The association between the UPS and mitochondria is further supported by the localization of UPS components in mitochondria. The USP30 deubiquitinating enzyme [41], and the E3 ubiquitin ligases Parkin [42], MARCHV/MITOL [43, 44], MAPL/MULAN [45], and RNF185 [46] localize at the OMM to mediate protein polyubiquitination. Interestingly, these UPS components' effects go beyond the clearance of damaged proteins and include the regulation of mitochondrial morphology and turnover. During ER stress, misfolded proteins accumulate into the ER lumen. They need to be retrotranslocated into the cytosol, where they are flagged with ubiquitin and degraded by the proteasome in a process called ER-associated protein degradation (ERAD) [47]. Mitochondria have an ERAD-like mechanism, the mitochondria-associated degradation (MAD) pathway, and share with ERAD some key components. These are the AAA ATPase p97, also referred to as valosin-containing protein (VCP), and the cofactor Npl4, both involved in the process of pulling out ubiquitinated proteins from the OMM and retrotranslocation to the cytosol [48]. p97 provides the driving force to extract Mfn1, Mfn2, and the anti-apoptotic protein MCL1 from the OMM and chaperone them to the proteasome [49, 50]. Interestingly, proteasome inhibition leads to accumulating the IMM proteins UCP2, COXI, III, IV, and OSCP [51]. One intriguing issue is whether intramitochondrial proteins that do not face the cytosol can be substrates of the MAD pathway. Even though retrotranslocation machinery has not been identified, some evidence indicates that the UPS controls IMM proteins. In fact, as a consequence of proximity to the respiratory chain, IMM proteins are exposed to ROS generated by mitochondrial respiration and, therefore, oxidized. For example, the mitochondrial matrix protein OSCP, a subunit of OXPHOS complex V, can be retrotranslocated to the OMM, where it can be ubiquitinated and degraded [51]. Moreover, unfolded IMS proteins can exit the IMS through the TOM translocase, which is the same route used for import [40].

#### Mitochondrial unfolding protein response (UPRmt)

Under stress conditions, when the degradation pathways are insufficient to repair the consequences of the alteration in mitochondrial translation [52] or the accumulation of oxidatively damaged or misfolded proteins, a retrograde signal is activated which coordinates nuclear gene expression. This mitochondria-to- nucleus response is named mitochondrial unfolding protein response (UPRmt) [53]. The ultimate purpose of UPRmt is to maintain proteostasis by promoting the expression of chaperones and m-AAA proteases ClpP and YME1L1, inside mitochondria. This pathway's activation improves protein folding, inhibits protein synthesis to alleviate ER stress, and removes damaged proteins. Thus, repairing and restoring mitochondrial proteostasis complex is critical for the adaptation to environmental changes that risk mitochondrial proteome integrity and, consequently, limit the mitochondrial damage.

### Mitochondrial dynamics

An essential mitochondrial quality control system is the dynamic remodeling of mitochondrial membranes, through repeated rounds of fusion and fission events. The continuous alternation of these two processes regulates mitochondrial number, morphology, and distribution, ensuring the adaptation of the mitochondrial network to the cellular bioenergetic requirements. Mitochondrial shape and function are strictly connected. Mitochondrial fusion leads to elongated organelles, which expand the mitochondrial network and its interconnectivity [32]. This process enables the redistribution of energy in the form of mitochondrial potential, metabolites, proteins, and mtDNA, improving calcium handling, and ATP synthesis [24, 54–57]. Besides, the fusion between healthy and damaged organelles allows to dilute the damaged material into the healthy network, avoids the accumulation of dysfunctional mitochondria, and maintains their overall function [58]. On the contrary, fission separates the dysfunctional or damaged components from the network. The resulting unconnected shorter mitochondria will be further removed via mitophagy. The rapid morphological adaptations, by balanced fusion and fission, are crucial to counteract defective components' accumulation within the mitochondrial network. However, due to defects in mitochondrial fusion, excessive fission generates isolated mitochondria that are less efficient in ATP production and are dysfunctional because they consume ATP to maintain their membrane potential [59]. Similarly, defects in the fission machinery can lead to a hyper fused mitochondrial network that hinders an efficient mitophagy process. The regulation of these processes is mediated by specific cellular proteins subjected to specific post-translational modifications that modify their function.

#### A specific machinery regulates cristae remodeling and the fusion of the outer and inner mitochondrial membrane

In mammals, mitochondrial fusion is independently regulated at the OMM and the IMM. It starts with the tethering of two adjacent mitochondria, continues with the fusion of OMM, and concludes with IMM fusion.

##### Mitofusins and OMM fusion

The OMM fusion is controlled by the OMM membrane-bound GTPases mitofusin 1 (MFN1) and mitofusin 2 (MFN2). MFN1 and MFN2 have a high degree of homology, but they do not have the same functions. Genetic loss of MFN1 induces a higher degree of mitochondrial fragmentation than MFN2 deletion [60]. This difference can be explained because MFN1 has a greater GTPase membrane tethering activity [61]. GTP hydrolysis of MFN1 causes a conformational change that allows the OMMs of opposing mitochondria to contact and fuse [61, 62]. Moreover, the role of MFN2 in the fusion process remains elusive and additional functions of Mfn2 have been reported. MFN2, and not MFN1, is expressed on the mitochondria-associated endoplasmic reticulum membranes (MAM) and, to a lesser extent, to the endoplasmic/sarcoplasmic reticulum (ER/SR) [63, 64]. This unique distribution allows for close communication between the two organelles. Indeed, MFN2 bridges mitochondria to ER/SR, facilitating critical processes linked to ER–mitochondria interactions like calcium homeostasis and the modulation of the UPR during ER stress via PERK [63, 65–68]. MFN2, like other mitochondria-shaping proteins, undergoes post-translational modifications. For instance, when PINK1 phosphorylates MFN2, it becomes a receptor for the ubiquitin ligase Parkin to activate mitophagy [69]. The E3 ligases HUWE1 also promotes MFN2 ubiquitination and proteasomal degradation [70]. Conversely, the formation of a Lys63–polyubiquitin chain by the E3 ligase MARCHV/MITOLl does not induce MFN2 degradation but increases its activity and MAM formation and function [71].

##### OPA1 and IMM fusion

Upon OMM fusion, the optic atrophy protein 1 (OPA1), a dynamin-like GTPase located in the IMM, is required for IMM fusion [72]. OPA1 deletion leads to mitochondrial fragmentation, whereas OPA1 overexpression induces mitochondrial elongation [73]. Post-translational modifications control mitochondrial fusion and dynamics. OPA1 activity is regulated by proteolytic processing. There are several splicing variants of OPA1 (eight in humans and four in mice), expressed in a tissue-specific manner. Some of them are also cleaved to generate soluble short OPA1 (OPA1S) from long OPA1 isoforms (OPA1L) [74].

The mitoproteases OMA1 and YME1L cleave OPA1 under different physiological conditions. OPA1L is anchored to the IMM by a transmembrane domain at the N-terminus and can be further cleaved in exon 5 (site S1) by OMA1, a process which depends on the mitochondrial membrane potential [75]. YME1L1 cuts the site S2 in exon 5b of a subset of OPA1L belonging to the splicing variants 4, 6, 7, and 8 [76] under high OXPHOS conditions [77]. OPA1S lacks the transmembrane domain and is soluble in the intermembrane space. OPA1-dependent mitochondrial fusion needs Mfn1 [73] as well as balanced OPA1S and OPA1L forms. However, under stress conditions, fusion can rely only on OPA1L, while OPA1S forms are dispensable [76]. Fusion is also enhanced by the interaction between OPA1L and the IMM lipid, cardiolipin [72]. On the contrary, the complete conversion of OPA1L into OPA1S inhibits fusion and increases mitochondrial fission [74, 76]. OPA1 is a pleiotropic protein that, by forming oligomers of soluble and membrane-bound OPA1 isoforms, also controls cristae remodeling and the assembly of respiratory chain complexes into supercomplexes, a structure that enhances mitochondrial respiration [78]. In addition, OPA1 activity and, consequently, mitochondrial dynamics are regulated by reversible lysine acetylation. Under stress conditions, hyperacetylation of OPA1 reduces its GTPase activity while SIRT3-dependent deacetylation increases OPA1 GTPase activity [79].

#### Mitochondrial fission

##### The central role of DRP1 in OMM fission

Mitochondrial fission is a multi-step process that depends primarily on the cytosolic GTPase dynamin-related protein 1 (DRP1). The association of mitochondria with the ER is required to identify the scission site in the mitochondrial network. Fission occurs at mitochondria–ER contact sites marked with mtDNA [80]. Here, ER tubules wrap around mitochondria and promote an initial reduction of the mitochondrial diameter in a process termed ER-associated mitochondrial division (ERMD) [81]. An initial constriction before DRP1action is necessary because the DRP1 spiral is narrower than the mitochondrial diameter. This process might be facilitated by the action of the ER protein inverted formin 2 (INF2), which induces actin polymerization at the ER–mitochondria contact sites, enabling force generation to drive initial mitochondrial constriction [82]. Importantly, ERMD increases in the presence of mtDNA. Thus, mtDNA synthesis is coupled to mitochondrial division to ensure the distribution of newly replicated mtDNA to daughter mitochondria [80]. Subsequently, DRP1 is recruited to the marked division sites to bind to its OMM receptors/adaptors. This binding facilitates DRP1 oligomerization, forming a ring-like structure that favors an additional narrowing of the membranes [83]. Also, GTP hydrolysis leads to a conformational change that further increases membrane constriction. Although DRP1 can tubulate the membranes, it cannot complete membrane scission [84]. Indeed, the GTPase Dynamin 2 (DYN2) assembles in the DRP1-mediated mitochondrial constriction neck to drive mitochondrial scission [85].

##### DRP1 adaptors

DRP1 lacks hydrophobic membrane-binding domains, and so, its recruitment depends on integral OMM proteins that act as receptors/adaptors. In yeasts, Fis1 acts as an adaptor to recruit to the OMM the DRP1 orthologue, DNM1, to the additional mitochondrial fission proteins MDV1 and CAV4 [86–88]. In mammals, there are no orthologues for MDV1 and Cav 4, and recent evidence suggests that Fis1 is not required for fission [89, 90]. Instead, it has been reported that other components of the mammalian fission bind to DRP1 and arrange the assembly of DRP1 oligomers at constriction sites: mitochondrial fission factor (MFF), mitochondrial elongation factor 2/mitochondrial dynamics protein 49 (MIEF2/MiD49), and MIEF1/MiD51 [90–93]. MFF overexpression results in increased mitochondrial fission [90, 93]. Selective recruitment of oligomerized forms of DRP1 to mitochondria, and stimulation of DRP1 GTPase activity [94]. The DRP1 affinity for MFF is higher than for Fis1, suggesting that MFF preferentially functions as a DRP1 receptor. On the contrary, silencing MFF generates elongated mitochondria and DRP1 cytosolic distribution [90, 93]. MIEF1/MiD51 and the variant MIEF2/MiD49 recruit DRP1 to mitochondria independent of MFF, but in contrast to MFF, inhibit DRP1 GTPase activity leading to mitochondrial elongation when MiD49/51 is overexpressed [92, 95, 96]. DRP1-dependent mitochondrial fission through MiD49 and/or MiD51, but not MFF, is required for cristae remodeling to facilitate cytochrome c release into the cytoplasm during apoptosis [97]. Although the DRP1 adaptors MiD49/51 and MFF are simultaneously expressed, each adaptor's activation might differ according to physiological circumstances since they have distinct roles in the modulation of DRP1-mediated mitochondrial fission.

##### The missing players of IMM fission

Mitochondrial division requires the division of both the inner and outer mitochondrial membranes. In the last fifteen years, we have further improved our understanding of the molecular components and regulation of the fission machinery, controlling OMM's scission. However, until now, little is known about the events leading to the IMM division. In prokaryotic cells and certain primitive eukaryotic species, FtsZ is a self-assembly GTPase that forms a calcium-mediated constricting ring on IMM's matrix side, suggesting that specific mechanisms for IMM constriction must exist [98]. However, mammals lack a homolog of the fission protein FtsZ [99]. In the absence of DRP1 or OMM constriction, the IMM division still occurs, indicating the presence of an inner membrane fission machinery independent of DRP1 [100, 101]. Recently, a Ca + 2-dependent mechanism for IMM constriction and division has been reported in neurons and human osteosarcoma cells. The inner membrane constriction occurs at ER–mitochondria contact sites before DRP1 recruitment and is thus independent of DRP1 action and OMM division [102, 103]. The mechanism which drives IMM constriction are not elucidated, but the requirement of MCU and OMA1-induced OPA1S has been shown [102, 103]. In line with the reports suggesting a link between mitochondrial calcium overload and mitochondrial fragmentation [104, 105], MCU inhibition leads to mitochondrial elongation [103]. Recent studies have underlined the role of OPA1S in the mitochondrial division [76]. Mitochondrial calcium overload leads to mitochondrial depolarization, leading to the OMA1-dependent processing of OPA1 in OPA1S. OPA1S accumulation disrupts the MICOS complex's capacity to stabilize OMM–IMM tethering, leading to IMM untethering and possible constriction [102]. Another player proposed to regulate the inner membrane fission is the IMM protein MTP18. Overexpression of MTP18 leads to mitochondrial fission, while its depletion causes hyperfusion of the mitochondrial network [106]. Moreover, MTP18 modulates DRP1 phosphorylation and mitochondrial recruitment downstream of the mTORC1 signaling pathway [107]. Further studies are required to identify the IMM fission machinery players and their regulation.

##### Regulation of the fission machinery

Several post-translational modifications like phosphorylation, ubiquitination, SUMOylation, S-nitrosylation, and O‐GlcNAcylation have been identified in DRP1 [108]. Phosphorylation of different residues of DRP1 can have either enhancing or inhibitory effects. For example, during mitosis, when organelles are inherited by daughter cells, Cdk1–cyclin B-dependent DRP1 phosphorylation at Ser616 (in humans) in the GTPase effector domain (GED), stimulates DRP1 oligomerization and thus, mitochondrial fission [109]. Phosphorylation of Ser637 by protein kinase A (PKA) inhibits DRP1 by promoting a cytosolic localization that leads to mitochondrial elongation [110]. During the early stages of starvation, increased phosphorylation of Ser637 due to PKA activity and decreased phosphorylation of Ser616 retain DRP1 in the cytosol, and thus, mitochondria are elongated and spared from autophagic degradation. The resulting tubular mitochondrial network displays an increased number and density of cristae and presents more dimers of the ATP synthase [55, 111]. DRP1 inhibition is counteracted by the calcium-dependent phosphatase, calcineurin, which drives DRP1 mitochondrial translocation and fission [110, 112]. DRP1 can also be ubiquitinated and targeted for proteasomal degradation by the E3 ligase Parkin [113], while the ubiquitination is mediated by MARCHV/MITOL have different outcomes depending on cell context [43, 114]. These controversial results might be explained by the fact that MFN1, MFN2, and MID49 are also substrates of MARCHV [71, 115, 116]. Moreover, DRP1 is not only a substrate but also a regulator of MARCHV activity along with MFF [117]. Thus, in addition to their canonical roles in mitochondrial fission, DRP1 and MFF might also act as regulatory factors that control mitochondrial fission and fusion. Also, MFF can be regulated by post-translational modifications. Upon mitochondrial dysfunction, MFF is phosphorylated by AMPK. This phosphorylation enhances DRP1 recruitment, mitochondrial fission, and mitophagy [118]. Conversely, the RNA-binding protein Pumilio 2 (PUM2) mediates a transcriptional inhibition of MFF, leading to impairment of mitochondrial fission, mitochondrial function, and mitophagy [119].

### Mitochondrial-derived vesicles (MDVs)—a lysosomal-dependent mechanism to repair mild mitochondrial damage

An alternative system complementary to mitophagy, for delivering mitochondrial misfolded or oxidized proteins and lipids to the lysosome for degradation, has been described. Mild mitochondrial damage, without global mitochondrial depolarization, activates the release from mitochondria of double-membrane vesicles, selectively enriched of oxidized OMM, IMM, and matrix proteins [120, 121]. Thus, oxidative stress triggers MDVs formation via the PINK1–Parkin system independent of mitochondrial fission and autophagy pathways [120, 122]. Although the identification of the machinery necessary for MDVs biogenesis is still unknown. These vesicles do not require DRP1 fission activity for MDVs budding [120], and their fusion with the lysosome depends on the OMM SNARE syntaxin 17 [123]. Therefore, MDVs function before canonical mitophagy to preserve the integrity of the organelle. In addition to the MDVs role in mitochondria quality control, the fusion between MDVs and ER-derived vesicles is critical for de novo peroxisomal biogenesis [124].

### Mitophagy—an autophagosome–lysosome mechanism for the entire degradation of irreversibly damaged mitochondria

The maintenance of an intact mitochondrial network requires the degradation of dysfunctional components and their replacement with new ones. Thus, the coordination between mitochondrial biogenesis and mitophagy processes regulates the constant turnover of the network. The activation of mitochondrial biogenesis pathways maintains an adequate mitochondrial pool by incorporating new components into the pre-existing mitochondrial reticulum. On the other hand, during mitophagy, irreversibly damaged organelles that have been flagged for degradation by specific proteins, are excised from the mitochondrial network by the fission machinery and sequestered into autophagic vesicles for their degradation in the lysosome. The loss of mitochondrial membrane potential is a major trigger for mitophagy [125].

#### Parkin-dependent mitophagy

In mammals, the best described mitophagy pathway is the one regulated by the proteins PINK1 and Parkin. Recessively inherited forms of Parkinson’s disease are associated with loss-of-function mutations of the PTEN-induced kinase 1 (PINK1) and the E3 ubiquitin ligase Parkin. Under basal conditions, PINK1 is imported into the IMM, which is cleaved by PARL in a voltage-dependent manner. The resulting fragments retro translocate from mitochondria to the cytosol, where they are further degraded by the UPS [38, 126]. Thus, healthy mitochondria have undetectable levels of PINK1. However, when the mitochondrial membrane potential is dissipated, full-length PINK1 is not further imported to the IMM and instead accumulates on OMM. Here, PINK1 is activated and phosphorylates at Ser65 of Parkin's ubiquitin-like domain, increasing its E3 ligase activity [127, 128]. PINK1 also phosphorylates at Ser65 the pre-existing ubiquitin molecules at the OMM, leading to further Parkin recruitment and activation [128]. Once phosphorylated, Parkin amplifies the mitophagy signal by building ubiquitin chains on OMM proteins to recruit the autophagy receptors on depolarized mitochondria [129]. The role of the autophagy receptors is to promote a bridge between the autophagosome and the ubiquitinated OMM protein. These receptors have a ubiquitin-binding domain that binds to the ubiquitin chains in the OMM and an LC3 Interacting Region (LIR) domain to interact with LC3 on the autophagosome. p62, optineurin, NDP52, and NRB1 receptors bind both ubiquitin and LC3 to initiate mitophagy [129]. In cells, p62/SQSTM1 is not required for mitophagy, but it is important for the perinuclear clustering of depolarized mitochondria [129, 130]. OPT and NDP52 are both required for mitophagy, but they have redundant roles [129]. However, these data were obtained in vitro, and thus, the physiological relevance in vivo needs to be validated. For instance, while p62 and NBR1 are well expressed in adult muscles, optineurin and NDP52 proteins are barely detectable [129, 131]. Recently, an inner membrane Parkin-dependent mitophagy receptor was identified. The IMM protein Prohibitin 2 (PHB2) promotes PINK1/Parkin-mediated mitophagy by decreasing PINK1 processing through PARL inhibition and the stabilization of PINK1 on the OMM through the action of mitochondrial serine/threonine-protein phosphatase PGAM5 [132]. Upon mitochondrial depolarization, Parkin mediates the recruitment of the proteasomes to damaged mitochondria, where they induce the rupture of the OMM [133, 134]. A proteasome-dependent outer membrane rupture is required for Parkin-mediated mitophagy. OMM rupture exposes PHB2 to the cytoplasmic environment, where it binds directly LC3 through a LIR domain via the cytosolic exposure of PHB2 [135]. The inhibition of the proteasome activity with epoxomicin prevents OMM rupture and the co-immunoprecipitation and colocalization of PHB2 with LC3, suggesting that OMM degradation precedes PHB2–LC3 interaction [135]. Importantly, PHB2 is required for the elimination of paternal mitochondrial DNA after embryonic fertilization in *C. elegans* [135]. However, basal mitophagy can also occur independent of the PINK1/Parkin pathway [136, 137]. Moreover, PINK1 and Parkin mice are viable and develop normally [138, 139]. Thus, the PINK1/Parkin mitophagy pathway could be compensated by other pathways during development and more generally during physiological mitophagy.

#### Parkin-independent mitophagy

Several constitutively expressed autophagy receptors are localized at the OMM and can interact via their LIR domains with the autophagosome protein LC3, triggering mitophagy independently of Parkin recruitment. The Parkin-independent mitophagy receptors are BNIP3, BNIP3L/NIX, FUNDC1, Bcl2-L-13, and AMBRA1. BNIP3 and BNIP3L/NIX are BH3-only proteins that are implicated in both apoptosis and mitophagy. These proteins translocate to mitochondria, form homodimers, and disrupt the mitochondrial membrane potential [140]. They contain two evolutionary conserved LIR domains, which can be post-translationally modified to regulate their interaction with LC3 and with the LC3 homologous protein, GABARAPL1. BNIP3 is phosphorylated on Ser 17 and 24, which promotes its interaction with LC3 [141]. The phosphorylation of BNIP3L/NIX on Ser 34 and 35 increases its interaction with GABARAPL1 [142]. Moreover, BNIP3 overexpression in cardiomyocytes requires DRP1 translocation to mitochondria to promote mitochondrial fission and mitophagy [141]. Besides removing damaged mitochondria, BNIP3L/NIX is also required for the selective mitochondrial elimination during reticulocyte differentiation [140, 144]. FUNDC1, like BNIP3 and BNIP3L/NIX, is tightly regulated by the phosphorylation of residues near the LIR domain. In basal conditions, FUNDC1 is inhibited by the phosphorylation on Tyr 18 by the Src kinase and Ser 13 by the kinase casein kinase 2 (CK2) [142, 143]. Upon stress, like hypoxia or mitochondrial uncoupling, the mitochondrial phosphatase PGAM5 removes the Ser 13 phosphorylation to allow FUNDC1 association with LC3B [142]. Moreover, the association of FUNDC1 with LC3 is further increased by ULK1-dependent phosphorylation on Ser 17 located in the LIR domain, [144]. The ubiquitination of FUNDC1 by the OMM resident ubiquitin E3 ligase MARCHV and its further proteasomal degradation is the mechanism that fine-tunes the mitophagy response under hypoxia [145]. Bcl2 like protein 13 (Bcl2-L-13), the mammalian homolog of Atg32, is localized in the outer mitochondrial membrane and induces mitochondrial fragmentation in the absence of Drp1 [146]. Bcl2-L-13 induces a Parkin-independent mitophagy through the interaction with LC3 via a conserved LIR motif [146]. Moreover, Bcl2-L-13 recruits the ULK1 complex, which binds to LC3 through a LIR domain [147]. The interconnection between Bcl2-L-13, ULK1, and LC3 is critical for Bcl2-L13-mediated mitophagy [147].

In summary, mitophagy works in conjunction with other mitochondria quality control pathways like mitochondrial proteolysis and dynamics.

## Mitochondria quality pathways are essential for skeletal muscle physiology

**Fig. 2:**
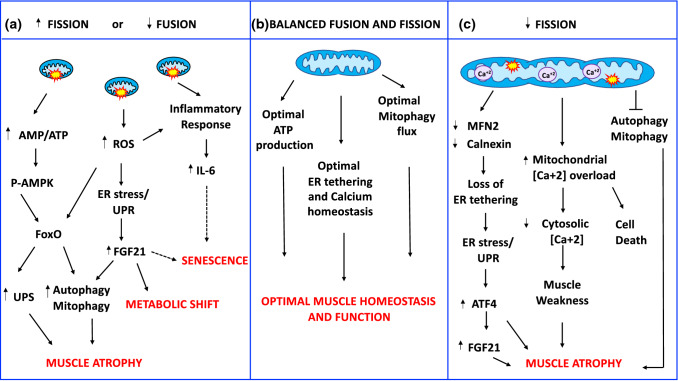
Mitochondria-derived signaling pathways controlling muscle mass and whole-body homeostasis. **a** Increased fission or decreased fusion leads to dysfunctional fragmented organelles, which activate the energy sensor AMPK by increasing the AMP/ATP ratio, ROS production, and the inflammatory response. P-AMPK directly phosphorylates FoxO3 increasing its transcriptional activity and affecting muscle mass. ROS production causes endoplasmic reticulum (ER) stress and activation of unfolded protein response (UPR). UPR induces the ATF4-dependent upregulation of FGF21 secreted by the muscle that contributes to muscle loss, causes a systemic metabolic shift, and premature senescence. **b** Balanced mitochondrial fusion and fission are critical for muscle function and whole-body homeostasis. **c** A reduction of mitochondrial fusion results in the accumulation of elongated dysfunctional mitochondria resulting in mitophagy impairment, loss of ER tethering, ER stress, increased mitochondrial calcium overload, and decreased cytosolic calcium causing cell death, muscle loss, and weakness. Dashed lines indicate mechanisms that need more studies

Skeletal muscle is a post-mitotic tissue. Its cells do not divide, and consequently, damaged/dysfunctional mitochondria cannot be diluted through cellular division. Therefore, post-mitotic tissues depend on the activation of coordinated pathways to preserve or restore mitochondrial function. The central role of mitochondria in skeletal muscle homeostasis depends not only on energy production, but also on the buffering of intracellular calcium, and the signaling pathways that control nuclear gene programs which regulate muscle mass. Alterations in mitochondrial distribution, morphology, and function are present in atrophic muscles in aging [148–150], muscle disuse [151, 152], burn injury [153], intensive care unit-acquired weakness [154], insulin resistance [155] chronic obstructive pulmonary disease (COPD) [156], cancer cachexia [157–159], and different neuromuscular disorders [160]. Over the last years, gain- and loss-of-function studies have further improved our understanding of the critical role of mitochondrial quality control pathways in regulating the nuclear programs controlling muscle loss (Fig. 2).

### Mitochondrial proteostasis is critical for skeletal muscle function

Different mechanisms degrade misfolded, aggregated, or damaged mitochondrial proteins and, thus, help avoid mitochondrial proteotoxicity (see paragraph 5.1). The coordinated action of specific mitochondrial proteases with the cytoplasmic UPS and a mitochondrial–nuclear communication stress response (UPRmt) culminates in repairing the organelles that are salvageable to yield a healthier mitochondrial network. These mechanisms are essential to keep under control the quality of the mitochondrial proteome. They are also critical for regulating mitochondrial dynamics, mitophagy, and apoptosis. The matrix mitochondrial protease LonP1 is necessary for the maintenance of mitochondrial matrix proteostasis. In mice, the homozygous deletion of LonP1 causes early embryonic death [161]. In human skeletal muscle, the reduction of LonP1 activity results in electron-dense intra-mitochondrial accumulations, mitochondrial dysfunction, oxidative stress, and muscle weakness [162–164].

#### Deletion of the mitoproteases involved in OPA1 processing in skeletal muscle

The accumulation of damaged proteins due to a homozygous missense mutation that impairs the IMS protease maturation, YME1L1, causes infantile onset mitochondriopathy with severe intellectual disability, muscular impairments, and optic nerve atrophy. Muscle biopsies from these patients display alterations in cristae morphology and paracrystalline inclusions and fiber-type grouping, indicating denervation [165]. In mice, total genetic ablation of Yme1L1 leads to embryonic death, while the specific inhibition of the gene in the heart causes dilated cardiomyopathy that shortens lifespan (median life span:46 weeks). Mechanistically, YME1L ablation in cardiomyocytes activates OMA1 and promotes OPA1 processing and mitochondrial fragmentation, which causes heart failure [166]. In agreement, double deletion of Yme1L1 and OMA1, specifically in the heart, restored mitochondrial morphology and rescued cardiac function [166]. Thus, Yme1L1 is essential for normal cardiac function. Interestingly, cardiac function and lifespan of mice lacking YME1L in cardiomyocytes are normalized without restoring mitochondrial morphology, when the deletion of YME1L happens in both cardiomyocytes and skeletal muscle fibers (median life span 125 weeks). This recovery is because the loss of YME1L in skeletal muscle and the consequent mitochondrial dysfunction triggers systemic glucose intolerance and lowers insulin levels preventing cardiac glucose overload and cardiomyopathy [166]. In contrast to Yme1L1, the germline deletion of OMA1 does not impair embryogenesis, which indicates that OMA1-induced OPA1 processing is dispensable for embryonic and adult mouse development [166, 167]. OMA1 deficiency causes the lack of processing of OPA1L, which results in the shift of mitochondrial dynamics towards fusion. OMA1-deficient mice display a diet-induced obesity phenotype, with increased hepatic steatosis and alteration of glucose metabolism, in addition to defective thermogenesis, suggesting a role for OMA1 in energy metabolism [167]. Likewise, also defects in PARL have been linked to metabolic dysfunction. PARL levels are reduced in skeletal muscle during aging and type 2 diabetes mellitus [168]. In mice, muscle knockdown of PARL results in an alteration in mitochondrial cristae morphology, mitochondrial dysfunction, oxidative stress, and altered insulin signaling, which probably can be explained by the reduction in PARL-mediated OPA1 processing [168]. The total genetic inactivation of PARL in mice supports this protease's important role in OPA1 processing and tissue homeostasis [175]. PARL-ablated mice display reduced levels of a soluble, intermembrane space (IMS) form of OPA1, which results in mitochondrial dysfunction, increased apoptosis, neurodegeneration, and progressive muscle wasting that shortens lifespan (median life span 10 weeks) [169].

#### p97/VCP and skeletal muscle mass

p97/VCP is critical in the retrotranslocation and degradation of endoplasmic reticulum-associated proteins (ERAD) [47] and mitochondrial proteins ( MAD) [49, 50]. Its gene disruption causes early embryonic lethality in mice, highlighting the importance of maintaining proteostasis during embryogenesis [170]. The relevance of mitochondrial retrotranslocation mechanism in quality control has been demonstrated by the direct link between p97/VCP mutation and human neuromuscular diseases, like Inclusion Body Myopathy, Frontotemporal dementia and Amyotrophic Lateral Sclerosis [171, 172]. Mutations in p97 in mice [173] and humans [174] cause mitochondrial swelling, cristae disruption, oxidative stress, and decreased ATP, resulting in skeletal muscle atrophy and weakness. Similarly, the inhibition of p97/VCP in zebrafish leads to mitochondrial morphology and cristae alterations in skeletal muscle and the heart [175]. Moreover, p97/VCP deficiency resulted in the accumulation of numerous vesicular bodies, autophagy impairment, and altered myofibrillar organization, causing a severe myopathy. Consequently, skeletal muscle [175], and cardiac function are impaired [175, 176]. In skeletal muscle, p97/VCP is critical in extracting ubiquitinated proteins from myofibrils during fasting- and denervation-induced atrophy. Interestingly, during muscle atrophy, induction of p97/VCP occurs when protein breakdown is maximal in myofibers [177].

In summary, the physiological role of the different mechanisms in charge of preserving mitochondrial proteostasis and thus, mitochondrial function is critical for skeletal muscle homeostasis and of muscle pathology control.

### Dysregulation of mitochondrial fusion in skeletal muscle leads to muscle atrophy, weakness, and have severe consequences in whole-body physiology

It was initially thought that because IMF mitochondria are densely packed between the myofibrils, communication and fusion between the surrounding organelles might be limited. This view changed when live-cell imaging experiments using photoswitchable probes showed that mitochondrial fusion occurs with rates that depend on the fiber's metabolic status. Mitochondrial fusion rates correlate with the OXPHOS capacity, higher in oxidative fibers than in glycolytic fibers [60]. Moreover,

mitochondrial fusion in skeletal muscle is necessary to adapt to the cell's specific functional needs and support skeletal muscle myofibers contractile function [54]. The full knockout mice of either Mfn1, Mfn2, or OPA1 result in embryonic lethality of mice, demonstrating the biological importance of mitochondrial fusion in early development [60, 178]. The relevance of mitochondrial fusion has also been highlighted in humans. Loss-of-function mutations in MFN2 and OPA1 genes cause two neurodegenerative diseases, Charcot–Marie–Tooth type 2A (CMT2A) [179] and dominant optic atrophy (DOA) [180, 181], respectively. CMT2A is an inherited neuropathy that is clinically characterized by muscle atrophy. OPA1 heterozygous missense recessive mutations cause DOA characterized by an aspecific myopathy with mitochondrial features [182, 183]. The first case of homozygous missense mutation has been recently reported in two sisters. They died at 2 and 10 months of age, showing myopathy, encephalopathy, and cardiomyopathy [184]. Therefore, mutations in fusion genes result in brain and muscle dysfunction. Accordingly, the reduction of the mitochondrial fusion machinery in muscle has been linked to age-related sarcopenia [185–187], and metabolic diseases like obesity and type 2 diabetes [188, 189] in both rodents and humans.

#### Inhibition of MFN1 and MFN2 in skeletal muscle

Muscle-specific simultaneous ablation of MFN1 and MFN2 induces profound muscle atrophy. The conditional deletion was generated using a Cre-recombinase that starts to be expressed in skeletal muscle during mice embryogenesis. Knockout mice are viable at birth, but they display muscle growth defects characterized by mitochondrial dysfunction, reduction of mitochondrial DNA (mtDNA) in skeletal muscle, and accumulation of point mutations deletions in the mitochondrial genome that cause death within 6–8 weeks of age [190]. Accordingly, inhibition of MFN1 GTPase activity leads to mitochondrial fragmentation and dysfunction that contribute to heart failure progression in mice [191]. MFN2 deletion in young muscle causes extensive mitochondrial fragmentation, mitochondrial dysfunction, ROS production, ER stress, and autophagy inhibition resulting in muscle atrophy [67, 186]. Importantly, MFN2 deficiency during aging is contributes to mitophagy flux inhibition and the accumulation of dysfunctional mitochondria driving age-associated metabolic alterations and sarcopenia [186].

#### OPA1 and the control of skeletal muscle homeostasis

##### OPA1 deletion

Inhibition of the IMM profusion factor, OPA1, in muscle resulted in a similar but more severe phenotype than skeletal muscle MFN1 and MFN2 ablation [187, 192, 193]. Deletion of OPA1 in adult mice (3–5 months of age) resulted in a decrease of mtDNA, reduced respiratory complexes and supercomplexes content, and complex activity, respiration, and mitochondrial membrane potential. When OPA1 was deleted during fetal development, animals died a few days after birth. Accordingly, acute OPA1 inhibition in adult animals' skeletal muscles triggers oxidative stress, ER stress, muscle atrophy, and weakness, recapitulating several features of the conditional model [187]. Moreover, OPA1-deficient muscles reverberate to the whole body, causing a systemic inflammatory response, senescence of epithelial tissues, and premature death. FGF21 mediates these pro-aging effects because the deletion of both OPA1 and FGF21 reverts precocious senescence and mortality [187]. Mechanistically, OPA1 inhibition causes mitochondrial dysfunction, ROS production, and mitochondrial DNA release that trigger different transcription factors such as FoxO3, NFkB, and ATF4 that coordinate the upregulation of atrophy-related genes, FGF21 and inflammatory cytokines like IL6. Altogether, these transcriptional-dependent programs enhance ubiquitin–proteasome and autophagy–lysosome protein breakdown, hypoglycemia, lipolysis, liver steatosis, inflammation, and a pro-senescent phenotype.

Interestingly, a mild inhibition of OPA1, which does not alter mitochondrial complex and supercomplex formation as well as mitochondrial DNA content and citrate synthase activity, in muscle during neonatal growth results in several beneficial metabolic changes in terms of resistance to obesity when the animals were challenged with a high-fat diet [193]. Consistent with the other OPA1 knockout mice, also in this model, the metabolic changes are mediated by the muscle secretion of FGF21, whose level was mildly increased when compared to the ones present in mice in which OPA1 is completely deleted in adulthood.

##### OPA1 overexpression

Further support in the role of OPA1 in the homeostatic control of muscle mass comes from the observations obtained with a genetic model of controlled OPA1 overexpression. OPA1 transgenic mice are protected from acute muscle loss induced by denervation [194] as well as from chronic muscle loss in a model of myopathy caused by muscle-specific deletion of the mitochondrial subunit COX15 [195].

Altogether, the maintenance of mitochondrial shape and function, through fusion events, is required not only to modulate nuclear gene expression programs and, thus, muscle mass but also to control the crosstalk of muscle with distant organs influencing the whole-body metabolic homeostasis.

### Mitochondrial fission controls muscle development, maintenance, and function; the link with ER stress and calcium homeostasis.

Mitochondrial fission is a quality control mechanism required to maintain a healthy mitochondrial network. Impairment of fission leads to the disruption of selective mitochondrial autophagic degradation, followed by dysfunctional organelles accumulation [58, 196–200]. Severe human disorders have been linked to defects in the fission machinery. Mutations in DRP1 lead to a severe neurological syndrome with microencephaly, hypotonia, alterations in brain development, and metabolism that cause neonatal lethality due to multi-system damage [201–203]. Patients with mutations in the DRP1 receptor MFF, suffer from developmental delay and acquired microcephaly, seizures, spasticity, and optic atrophy [204, 205]. Loss-of-function mutations in MiD49 cause severe myopathy in humans, with Complex I and Complex IV deficiency in muscle [206]. In mice, MFF deletion leads to smaller animals that display neuromuscular defects, kyphosis, and premature death at 13 weeks due to dilated cardiomyopathy [207].

#### DRP1 and muscle atrophy

Constitutive DRP1 knockout animals are embryonically lethal, demonstrating that the fission machinery is critical for tissue development and function [208, 209]. Consistent with this crucial role, conditional ablation of DRP1 in the heart, brain, and skeletal muscle causes lethality [196, 198–200, 208, 209]. Disrupted mitochondrial fission, obtained with the specific ablation of DRP1 in the heart, induces accumulation of defective mitochondria due to impaired autophagy/mitophagy, that over-time promotes cardiomyocyte death [198–200]. There is strong evidence in skeletal muscle that supports a causal link between the dysregulation of mitochondrial fission and alterations in muscle maintenance [152, 196, 197, 210, 211].

##### DRP1 overexpression

Acute overexpression of DRP1 is sufficient to activate mitochondrial dysfunction, mitophagy, and energy stress, which result in the activation of an atrophy program via the AMPK–FoxO3 axis [152]. Accordingly, the constitutive overexpression of DRP1 in skeletal muscle caused muscle loss and decreased exercise performance. In this mouse model, stress-induced mitochondria-dependent signals activate both the UPRmt and the eIF2α–ATF4– FGF21 axis, causing a reduction in protein synthesis and a blockade of growth hormones actions that prevent muscle growth [210]. Of note, FGF21 overexpression in skeletal muscle induces BNIP3-dependent mitophagy and muscle atrophy [212].

##### DRP1 ablation

We have recently explored the physiological relevance of DRP1 in skeletal muscle homeostasis by generating two muscle-specific DRP1-null mouse models. Early deletion of DRP1 in skeletal muscle during embryogenesis resulted in reduced postnatal growth and premature lethality, while its acute ablation in adulthood causes muscle loss and degeneration [196]. Mechanistically, DRP1 inhibition induced autophagy and mitophagy impairment, MCU upregulation and mitochondrial calcium overload, ER stress, UPR activation, and FGF21 induction. ER stress is induced by the decrease of the ER–mitochondria tethering protein Mfn2 and the down- and upregulation of the ER chaperones calnexin and Bip/Grp78, respectively [63, 65, 66, 213]. Increased FGF21 levels can explain the observed metabolic changes, such as basal hypoglycemia, liver GH resistance, and conditional knockout mice's reduced animal size. In DRP1-null muscles, the sarcoplasmic reticulum's calcium stores are unchanged, while abnormal elongated mitochondria display increased MCU-dependent mitochondrial Ca2 + uptake capacity, leading to myofiber death and muscle regeneration [196]. Another study using muscle-specific DRP1 heterozygote mice showed reduced muscle endurance and running performance, and altered muscle adaptations in response to exercise training [211].

#### MFF regulation and muscle mass

A recent report investigated the physiological role of the post-transcriptional regulation of MFF in skeletal muscle. The RNA-binding protein PUM2 binds and represses, specifically the translation of MFF mRNA [119]. PUM2 levels increase with age in worms, mice and humans, while MFF is reduced upon aging, suggesting that abnormal mitochondrial fission and mitophagy contribute to age-related sarcopenia. Accordingly, the specific deletion of PUM2 in old mice's skeletal muscle increases MFF levels, enhances mitochondrial fission and mitophagy, and improves mitochondrial function and lifespan [119]. In line with this report, DRP1 overexpression in muscles of Drosophila slows aging sarcopenia by ameliorating mitochondrial morphology, function, and mitophagy [214].

Thus, mitochondrial fission is critical for muscle mass maintenance and homeostasis and can be protective or detrimental according to the degree of induction and the physiological context.

### The balance between fusion and fission events is critical for muscle mass and whole-body homeostasis.

Muscle loss in aging sarcopenia [185, 187, 215], cancer cachexia, chemotherapy-induced cachexia [157–159], and in a model of myasthenia gravis [216], is characterized by the decline of both fusion and fission machinery. Under physiological conditions, fusion and fission processes are balanced to control mitochondrial morphology, size, and number. This equilibrium can transiently change to meet the metabolic needs of the cell. However, an excessive activation or the impairment of either fusion or fission alter the balance and compromise mitochondrial function and cell health. Several reports have shown that rebalancing of mitochondrial dynamics rescues the phenotype of certain diseases associated with alterations of mitochondrial fusion or fission [119, 194, 195, 214], thus raising the question of whether it is more important the proper balance or the absolute levels of mitochondrial fusion and fission events. For instance, the specific deletion of MFN1, MFN2, and DRP1 leads to less severe cardiomyopathy and delays mortality with respect to unopposed fission or fusion [217]. Similarly, the simultaneous deletion of MFN1 and MFF in mice rescues mitochondrial function, heart dysfunction, and lifespan of both lethal MFN1 and MFF knockout mice [207]. Acute muscle-specific ablation of OPA1 and DRP1 (DKO) [197] shows a less severe phenotype when compared to OPA1 knockout mice. DKO showed muscle loss and weakness due to FoxO-dependent activation of the ubiquitin–proteasome system and general autophagy impairment. As a consequence of mitochondrial dysfunction, ER stress, UPR, and FGF21 pathways are activated and further contribute to muscle atrophy. The atrophy program's initial activation and the induction of FGF21 resolve over time in DKO muscles despite persistent mitochondrial dysfunction. Moreover, muscle denervation, oxidative stress, and inflammation are mitigated in DKO muscles, rescuing the lethal phenotype of OPA1 knockout mice [197]. In conclusion, unbalanced mitochondrial dynamics are more deleterious than the simultaneous reduction of fusion and fission processes. Therefore, it is possible that in case of a profound inhibition of either fusion or fission machinery, muscle cells downregulate the other one to mitigate the detrimental effects of an unbalanced mitochondrial dynamics. This compensatory effect would explain why both fusion and fission can be reduced simultaneously in different catabolic conditions.

### FGF21 and the inhibition of fusion, fission, or both in skeletal muscle—a common factor with different outcomes

As stated before, muscle-specific OPA1, DRP1, and the double OPA1/DRP1 (DKO) knockout mice have common alterations in mitochondrial function, ER stress, and UPS activation but different phenotypes in terms of muscle atrophy, weakness, senescence and animal survival (Table 1). Interestingly, the stress-response myokine FGF21 is dramatically increased in muscle and serum of all these three models, even if with a different degree. FGF21 serum levels are lower in DRP1 knockout mice than OPA1-null mice. Since FGF21 effects are dose dependent, a moderate induction results in adaptive responses to stress, while a dramatic increase is detrimental [218]. Another possibility explains the difference in the phenotypes, the induction of the inflammatory response might synergize with FGF21 in senescence induction. OPA1 inhibition triggers IL6 and IL1 upregulation via ROS [187], while the ablation of DRP1 does not alter the expression of inflammatory cytokines [196]. Consistently, the simultaneous inhibition of OPA1 and DRP1 rescues mortality [197] and FGF21 serum levels are only transiently elevated. Notably, the decrease of FGF21 levels correlated with a partial muscle mass recovery [212]. Thus, FGF21 is commonly induced when mitochondrial function is impaired and dictates healthy or unhealthy skeletal muscle outcomes. The effects of FGF21 will result from the combination of several factors, including the presence of synergizing or antagonizing factors; FGF21 blood level that reaches a certain threshold can elicit adverse effects and acute and transient versus chronic and persistent FGF21 secretion. According to the combination of these variables, FGF21 can be both a therapeutic agent and a biomarker of disease [219].

**Table 1:**
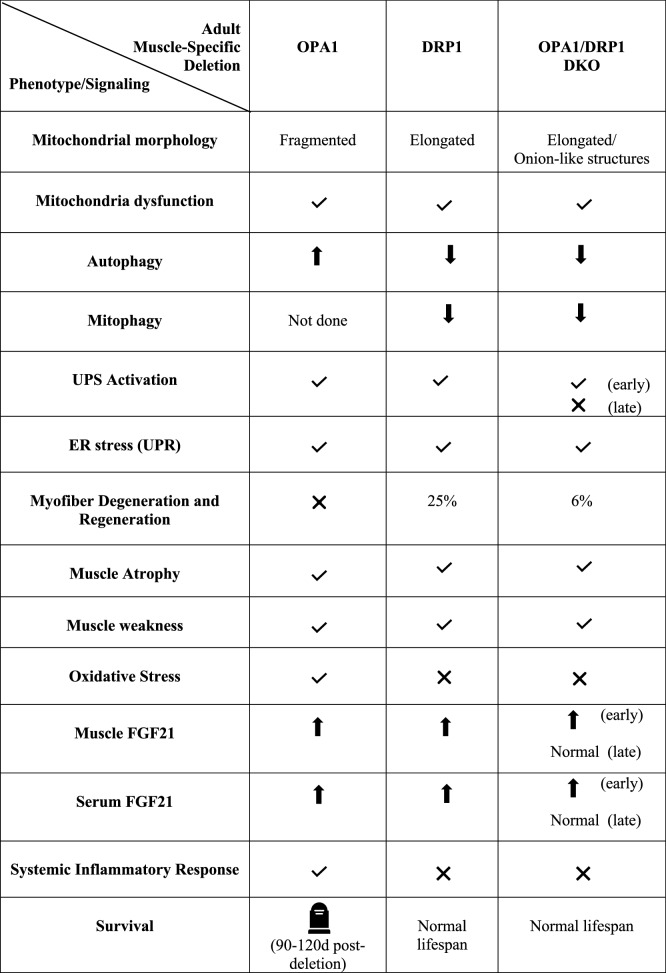
Signaling and phenotype of muscle-specific of Opa1-, Drp-, and Drp1/Opa1-null mice

Early: 70 days post-deletion, late: 365 days post-deletion.

### Balanced mitophagy flux is a crucial regulator of muscle mass and homeostasis

Mitophagy is a cellular housekeeping mechanism to keep under control the mitochondrial network in both physiological conditions and in response to cellular stress. For example, during starvation, the activation of autophagy is a fundamental survival mechanism that ensures optimal energy utilization. The protective role of the autophagy pathway is most evident immediately after birth when the transplacental nutrient supply is interrupted. Indeed, mice deficient for autophagy essential genes, such as ATG5, or ATG7, die soon after birth [220, 221].

#### Exercise and autophagy in skeletal muscle

Energy stress consequent to acute or chronic exercise triggers autophagy in different tissues [197–200]. The acute deletion of ATG7, specifically in skeletal muscle, does not affect exercise performance suggesting that autophagy is not required to sustain muscle contraction during exercise training [222, 223]. However, the activation of autophagy during exercise is important for maintaining muscle energy homeostasis. Moreover, mitophagy activation removes dysfunctional mitochondria that have been altered by exercise-dependent ROS production [222].

#### Muscle mass depends on the fine-tuning of the autophagy and mitophagy flux

The mechanisms regulating mitophagy during exercise have been recently reviewed in [224]. Importantly, the protective effects of autophagy and mitophagy rely on the fine-tuning of the flux; otherwise, it can become detrimental instead of being protective. Both excessive autophagy [140, 186] and the deficient degradation of cytosolic components [225, 226] contribute to muscle atrophy. The activation of mitophagy triggered by the transient overexpression of BNIP3 and BNIP3L induce muscle atrophy [15, 152]. In agreement, transient inhibition of BNIP3 in skeletal muscle partially protects from muscle loss induced by fasting, FoxO3, and FGF21 overexpression [15, 152, 212]. Conversely, reduction of mitophagy caused by the ablation of PINK and Parkin induces mitochondrial dysfunction and increased sensitivity to oxidative stress followed by muscle degeneration [225–228]. Accordingly, skeletal muscle-specific casein kinase 2 (CK2) deletion leads to a block of the autophagy and mitophagy flux that results in myopathy and muscle weakness [229]. The reduced CK2-mediated TOMM22 phosphorylation weakens the binding between PINK1 and TOMM22, leading to decreased IMM PINK1 import and further OMM PINK1 accumulation. Consequently, there is an increase in autophagosome formation on mitochondria, that however, cannot fuse with the lysosome resulting in mitophagy impairment and mitochondrial dysfunction during the time [229]. Likewise, impairment of autophagy by muscle-specific ablation of ATG5 and ATG7 induces accumulation of abnormal mitochondria, induction of oxidative stress, apoptosis, muscle atrophy, weakness, several features of myopathy [230, 231]. Also, it exacerbates fasting- and denervation-induced atrophy [231]. Accordingly, inhibition of autophagy by Parkin inhibition exacerbates statin-induced myopathy [232]. Similarly, in sarcopenia, the age-related loss of muscle mass, there is a progressive accumulation of macromolecules and dysfunctional mitochondria due to a decline of both general autophagy and mitophagy [233]. Accordingly, muscle-specific ATG7 null mice display premature aging characterized by increased oxidative stress, mitochondrial dysfunction, muscle loss and weakness, and degeneration of neuromuscular junctions [234]. Further indications of the role of mitophagy in the maintenance of muscle homeostasis and neuromuscular junctions, comes from a paper investigating the role of mTORC1 in adult skeletal muscle [235]. Long-term mTORC1 inhibition in muscles results in myopathy, muscle weakness, and alterations in neuromuscular junctions reducing general autophagy and mitophagy. Reactivation of the autophagy and mitophagy flux with the autophagy activating peptide Tat-beclin1 is sufficient to prevent mitochondrial dysfunction and fiber denervation [235]. Boosting mitophagy by overexpressing PINK1, Parkin, DRP1, or p62 in Drosophila muscles improves the age-dependent muscle function deterioration and extends lifespan [214, 236, 237]. Moreover, exercise is the best non-pharmacological strategy to reactivate mitophagy, which reduces the accumulation of ROS and thus, improves mitochondrial health and delays the loss of muscle mass that accompanies various pathologies, including aging sarcopenia, heart failure and neurogenic myopathy [224, 238–240]. Thus, the selective removal of defective mitochondria via mitophagy is critical to preserve muscle function.

#### Alterations of the autophagy flux and myopathy

Dysregulation of the autophagy flux is detrimental for myofiber health and is a common feature of several myopathies [241]. The autophagic vacuolar myopathies (AVMs) are a group of muscle disorders characterized by the accumulation of autophagosome vesicles in muscles due to alterations in proteins involved in lysosomal acidification, lysosomal degradation of glycogen, and the maturation and fusion of autophagosomes that result in defective autophagy. AVMs include X-linked myopathy with excessive autophagy (XMEA), Danon disease (DD), and Pompe/glycogen storage disease type II (GSD II) [241]. VPS15 is a PI3 kinase regulator involved in autophagosome maturation. Muscle-specific VPS15 knockout mice develop a severe myopathy with hallmarks of DD myopathy, including alterations in mitochondrial morphology, and accumulation of autophagosomes and glycogen due to a defect in the fusion of autophagosomes with lysosomes. Also, over-expression of the VPS34–VPS15 complex in Danon disease patients in myoblasts results in a partial amelioration of glycogen overload [242]. Defective autophagy plays a role in congenital muscular dystrophies caused by defects in collagen VI production [243], laminin A/C [244] or dystrophin [245]. These dystrophic models have, in common, hyperactivation of the Akt/mTOR signaling pathway that inhibits autophagy. Dystrophic muscles present the accumulation of structurally altered mitochondria together with myofiber degeneration. Importantly, autophagy flux reactivation by dietary or pharmacological tools, like rapamycin, cyclosporine A, or AICAR, rescues the dystrophic phenotype by clearing the abnormal mitochondria [243–246]. Altogether, the regulation of a finely tuned mitophagy flux is central in preventing mitochondrial dysfunction, denervation, and weakness during aging and in several pathological conditions.

## Conclusions and perspectives

Mitochondria undergo adaptive structural and functional remodeling to meet the dynamic changes in the cell's metabolic demands. Multiple mechanisms have evolved to couple the constant reshaping of the mitochondrial network to the regulation of mitochondrial function, including the activation of proteolytic cascades, mitochondrial fusion, and fission mitophagy. These mechanisms collectively constitute an interconnected mitochondrial quality control system that recognizes and resolves mitochondrial dysfunction, crucial for skeletal muscle mass maintenance. Dysregulation in any of these mechanisms triggers catabolic signaling pathways, which feed-forward to the nucleus to promote the activation of muscle atrophy. A deeper understanding of these signaling pathways is required to identify pharmacological targets that modulate mitochondrial function and prevent muscle loss.

Moreover, skeletal muscle is an endocrine organ that releases myokines in response to mitochondrial stress, such as FGF21, regulating the physiology of the organism, and influencing disease progression in other tissues. Thus, mitochondria are signaling platforms that not only control muscle mass, but also mediate systemically the communication of the muscle with distant tissues influencing the whole-body homeostasis. The complete identification of the myokines, which are released by muscle in response to exercise and disease, as well as unraveling their mechanism of action, is crucial to provide therapeutic applications in age-related diseases.

## References

[1] Srikanthan P, Karlamangla AS (2014). Muscle mass index as a predictor of longevity in older adults. Am J Med.

[2] Newman AB, Kupelian V, Visser M (2006). Strength, but not muscle mass, is associated with mortality in the health, aging and body composition study cohort. J Gerontol A Biol Sci Med Sci.

[3] Gaitanos GC, Williams C, Boobis LH, Brooks S (1993). Human muscle metabolism during intermittent maximal exercise. J Appl Physiol.

[4] Sartori R, Milan G, Patron M (2009). Smad2 and 3 transcription factors control muscle mass in adulthood. Am J Physiol Cell Physiol.

[5] Trendelenburg AU, Meyer A, Rohner D (2009). Myostatin reduces Akt/TORC1/p70S6K signaling, inhibiting myoblast differentiation and myotube size. Am J Physiol Cell Physiol.

[6] Sartori R, Schirwis E, Blaauw B (2013). BMP signaling controls muscle mass. Nat Genet.

[7] Winbanks CE, Chen JL, Qian H (2013). The bone morphogenetic protein axis is a positive regulator of skeletal muscle mass. J Cell Biol.

[8] Sartori R, Sandri M (2015). Bone and morphogenetic protein signalling and muscle mass. Curr Opin Clin Nutr Metab Care.

[9] Sandri M, Sandri C, Gilbert A (2004). Foxo transcription factors induce the atrophy-related ubiquitin ligase atrogin-1 and cause skeletal muscle atrophy. Cell.

[10] Bodine SC, Latres E, Baumhueter S (2001). Identification of ubiquitin ligases required for skeletal muscle atrophy. Science.

[11] Lecker SH, Jagoe RT, Gilbert A (2004). Multiple types of skeletal muscle atrophy involve a common program of changes in gene expression. FASEB J.

[12] Gomes MD, Lecker SH, Jagoe RT (2001). Atrogin-1, a muscle-specific F-box protein highly expressed during muscle atrophy. Proc Natl Acad Sci USA.

[13] Sacheck JM, Hyatt J-PK, Raffaello A (2007). Rapid disuse and denervation atrophy involve transcriptional changes similar to those of muscle wasting during systemic diseases. FASEB J.

[14] Taillandier D, Polge C (2019). Skeletal muscle atrogenes: from rodent models to human pathologies. Biochimie.

[15] Mammucari C, Milan G, Romanello V (2007). FoxO3 controls autophagy in skeletal muscle in vivo. Cell Metab.

[16] Milan G, Romanello V, Pescatore F (2015). Regulation of autophagy and the ubiquitin-proteasome system by the FoxO transcriptional network during muscle atrophy. Nat Commun.

[17] Judge SM, Wu C-L, Beharry AW (2014). Genome-wide identification of FoxO-dependent gene networks in skeletal muscle during C26 cancer cachexia. BMC Cancer.

[18] Brocca L, Toniolo L, Reggiani C (2017). FoxO-dependent atrogenes vary among catabolic conditions and play a key role in muscle atrophy induced by hindlimb suspension. J Physiol (Lond).

[19] Ebert SM, Monteys AM, Fox DK (2010). The transcription factor ATF4 promotes skeletal myofiber atrophy during fasting. Mol Endocrinol.

[20] Ebert SM, Dyle MC, Kunkel SD (2012). Stress-induced skeletal muscle Gadd45a expression reprograms myonuclei and causes muscle atrophy. J Biol Chem.

[21] Vincent AE, White K, Davey T (2019). Quantitative 3D mapping of the human skeletal muscle mitochondrial network. Cell Rep.

[22] Vendelin M, Béraud N, Guerrero K (2005). Mitochondrial regular arrangement in muscle cells: a “crystal-like” pattern. Am J Physiol, Cell Physiol.

[23] Ferreira R, Vitorino R, Alves RMP (2010). Subsarcolemmal and intermyofibrillar mitochondria proteome differences disclose functional specializations in skeletal muscle. Proteomics.

[24] Glancy B, Hartnell LM, Malide D (2015). Mitochondrial reticulum for cellular energy distribution in muscle. Nature.

[25] Romanello V, Sandri M (2013). Mitochondrial biogenesis and fragmentation as regulators of protein degradation in striated muscles. J Mol Cell Cardiol.

[26] Dahl R, Larsen S, Dohlmann TL (2015). Three-dimensional reconstruction of the human skeletal muscle mitochondrial network as a tool to assess mitochondrial content and structural organization. Acta Physiol.

[27] Picard M, McManus MJ, Csordás G (2015). Trans-mitochondrial coordination of cristae at regulated membrane junctions. Nat Commun.

[28] Glancy B, Hartnell LM, Combs CA (2017). Power grid protection of the muscle mitochondrial reticulum. Cell Rep.

[29] Vincent AE, Turnbull DM, Eisner V (2017). Mitochondrial nanotunnels. Trends Cell Biol.

[30] Schiaffino S, Reggiani C (2011). Fiber types in mammalian skeletal muscles. Physiol Rev.

[31] Mishra P, Varuzhanyan G, Pham AH, Chan DC (2015). Mitochondrial dynamics is a distinguishing feature of skeletal muscle fiber types and regulates organellar compartmentalization. Cell Metab.

[32] Bleck CKE, Kim Y, Willingham TB, Glancy B (2018). Subcellular connectomic analyses of energy networks in striated muscle. Nat Commun.

[33] Lightowlers RN, Rozanska A, Chrzanowska-Lightowlers ZM (2014). Mitochondrial protein synthesis: figuring the fundamentals, complexities and complications, of mammalian mitochondrial translation. FEBS Lett.

[34] Calvo SE, Clauser KR, Mootha VK (2016). MitoCarta2.0: an updated inventory of mammalian mitochondrial proteins. Nucleic Acids Res.

[35] Grevel A, Pfanner N, Becker T (2019). Coupling of import and assembly pathways in mitochondrial protein biogenesis. Biol Chem.

[36] Vincow ES, Thomas RE, Merrihew GE (2019). Autophagy accounts for approximately one-third of mitochondrial protein turnover and is protein selective. Autophagy.

[37] Ahola S, Langer T, MacVicar T (2019). Mitochondrial proteolysis and metabolic control. Cold Spring Harb Perspect Biol.

[38] Jin SM, Lazarou M, Wang C (2010). Mitochondrial membrane potential regulates PINK1 import and proteolytic destabilization by PARL. J Cell Biol.

[39] Jeon HB, Choi ES, Yoon JH (2007). A proteomics approach to identify the ubiquitinated proteins in mouse heart. Biochem Biophys Res Commun.

[40] Bragoszewski P, Gornicka A, Sztolsztener ME, Chacinska A (2013). The ubiquitin-proteasome system regulates mitochondrial intermembrane space proteins. Mol Cell Biol.

[41] Nakamura N, Hirose S (2008). Regulation of mitochondrial morphology by USP30, a deubiquitinating enzyme present in the mitochondrial outer membrane. MBoC.

[42] Narendra D, Tanaka A, Suen D-F, Youle RJ (2008). Parkin is recruited selectively to impaired mitochondria and promotes their autophagy. J Cell Biol.

[43] Nakamura N, Kimura Y, Tokuda M (2006). MARCH-V is a novel mitofusin 2- and Drp1-binding protein able to change mitochondrial morphology. EMBO Rep.

[44] Yonashiro R, Ishido S, Kyo S (2006). A novel mitochondrial ubiquitin ligase plays a critical role in mitochondrial dynamics. EMBO J.

[45] Li W, Bengtson MH, Ulbrich A (2008). Genome-wide and functional annotation of human E3 ubiquitin ligases identifies MULAN, a mitochondrial E3 that regulates the organelle’s dynamics and signaling. PLoS ONE.

[46] Tang F, Wang B, Li N (2011). RNF185, a novel mitochondrial ubiquitin E3 ligase, regulates autophagy through interaction with BNIP1. PLoS ONE.

[47] Ye Y, Meyer HH, Rapoport TA (2001). The AAA ATPase Cdc48/p97 and its partners transport proteins from the ER into the cytosol. Nature.

[48] Heo J-M, Livnat-Levanon N, Taylor EB (2010). A stress-responsive system for mitochondrial protein degradation. Mol Cell.

[49] Tanaka A, Cleland MM, Xu S (2010). Proteasome and p97 mediate mitophagy and degradation of mitofusins induced by Parkin. J Cell Biol.

[50] Xu S, Peng G, Wang Y (2011). The AAA-ATPase p97 is essential for outer mitochondrial membrane protein turnover. Mol Biol Cell.

[51] Margineantu DH, Emerson CB, Diaz D, Hockenbery DM (2007). Hsp90 inhibition decreases mitochondrial protein turnover. PLoS ONE.

[52] Callegari S, Dennerlein S (2018). Sensing the stress: a role for the UPRmt and UPRam in the quality control of mitochondria. Front Cell Dev Biol.

[53] Zhao Q, Wang J, Levichkin IV (2002). A mitochondrial specific stress response in mammalian cells. EMBO J.

[54] Eisner V, Lenaers G, Hajnóczky G (2014). Mitochondrial fusion is frequent in skeletal muscle and supports excitation-contraction coupling. J Cell Biol.

[55] Gomes LC, Di Benedetto G, Scorrano L (2011). During autophagy mitochondria elongate, are spared from degradation and sustain cell viability. Nat Cell Biol.

[56] Skulachev VP (2001). Mitochondrial filaments and clusters as intracellular power-transmitting cables. Trends Biochem Sci.

[57] Ono T, Isobe K, Nakada K, Hayashi JI (2001). Human cells are protected from mitochondrial dysfunction by complementation of DNA products in fused mitochondria. Nat Genet.

[58] Twig G, Elorza A, Molina AJA (2008). Fission and selective fusion govern mitochondrial segregation and elimination by autophagy. EMBO J.

[59] Benard G, Bellance N, James D (2007). Mitochondrial bioenergetics and structural network organization. J Cell Sci.

[60] Chen H, Detmer SA, Ewald AJ (2003). Mitofusins Mfn1 and Mfn2 coordinately regulate mitochondrial fusion and are essential for embryonic development. J Cell Biol.

[61] Ishihara N, Eura Y, Mihara K (2004). Mitofusin 1 and 2 play distinct roles in mitochondrial fusion reactions via GTPase activity. J Cell Sci.

[62] Cao Y-L, Meng S, Chen Y (2017). MFN1 structures reveal nucleotide-triggered dimerization critical for mitochondrial fusion. Nature.

[63] de Brito OM, Scorrano L (2008). Mitofusin 2 tethers endoplasmic reticulum to mitochondria. Nature.

[64] Naon D, Zaninello M, Giacomello M (2016). Critical reappraisal confirms that Mitofusin 2 is an endoplasmic reticulum-mitochondria tether. Proc Natl Acad Sci USA.

[65] Muñoz JP, Ivanova S, Sánchez-Wandelmer J (2013). Mfn2 modulates the UPR and mitochondrial function via repression of PERK. EMBO J.

[66] Ngoh GA, Papanicolaou KN, Walsh K (2012). Loss of mitofusin 2 promotes endoplasmic reticulum stress. J Biol Chem.

[67] Sebastián D, Hernández-Alvarez MI, Segalés J (2012). Mitofusin 2 (Mfn2) links mitochondrial and endoplasmic reticulum function with insulin signaling and is essential for normal glucose homeostasis. Proc Natl Acad Sci USA.

[68] Ainbinder A, Boncompagni S, Protasi F, Dirksen RT (2015). Role of Mitofusin-2 in mitochondrial localization and calcium uptake in skeletal muscle. Cell Calcium.

[69] Chen Y, Dorn GW (2013). PINK1-phosphorylated mitofusin 2 is a Parkin receptor for culling damaged mitochondria. Science.

[70] Leboucher GP, Tsai YC, Yang M (2012). Stress-induced phosphorylation and proteasomal degradation of mitofusin 2 facilitates mitochondrial fragmentation and apoptosis. Mol Cell.

[71] Sugiura A, Nagashima S, Tokuyama T (2013). MITOL regulates endoplasmic reticulum-mitochondria contacts via Mitofusin2. Mol Cell.

[72] Ban T, Ishihara T, Kohno H (2017). Molecular basis of selective mitochondrial fusion by heterotypic action between OPA1 and cardiolipin. Nat Cell Biol.

[73] Cipolat S, Martins de Brito O, Dal Zilio B, Scorrano L (2004). OPA1 requires mitofusin 1 to promote mitochondrial fusion. Proc Natl Acad Sci USA.

[74] Ishihara N, Fujita Y, Oka T, Mihara K (2006). Regulation of mitochondrial morphology through proteolytic cleavage of OPA1. EMBO J.

[75] Head B, Griparic L, Amiri M (2009). Inducible proteolytic inactivation of OPA1 mediated by the OMA1 protease in mammalian cells. J Cell Biol.

[76] Anand R, Wai T, Baker MJ (2014). The i-AAA protease YME1L and OMA1 cleave OPA1 to balance mitochondrial fusion and fission. J Cell Biol.

[77] Mishra P, Carelli V, Manfredi G, Chan DC (2014). Proteolytic cleavage of Opa1 stimulates mitochondrial inner membrane fusion and couples fusion to oxidative phosphorylation. Cell Metab.

[78] Cogliati S, Frezza C, Soriano ME (2013). Mitochondrial cristae shape determines respiratory chain supercomplexes assembly and respiratory efficiency. Cell.

[79] Samant SA, Zhang HJ, Hong Z (2014). SIRT3 deacetylates and activates OPA1 to regulate mitochondrial dynamics during stress. Mol Cell Biol.

[80] Lewis SC, Uchiyama LF, Nunnari J (2016). ER-mitochondria contacts couple mtDNA synthesis with mitochondrial division in human cells. Science.

[81] Friedman JR, Lackner LL, West M (2011). ER tubules mark sites of mitochondrial division. Science.

[82] Korobova F, Ramabhadran V, Higgs HN (2013). An actin-dependent step in mitochondrial fission mediated by the ER-associated formin INF2. Science.

[83] Kraus F, Ryan MT (2017). The constriction and scission machineries involved in mitochondrial fission. J Cell Sci.

[84] Yoon Y, Pitts KR, McNiven MA (2001). Mammalian dynamin-like protein DLP1 tubulates membranes. Mol Biol Cell.

[85] Lee JE, Westrate LM, Wu H (2016). Multiple dynamin family members collaborate to drive mitochondrial division. Nature.

[86] Mozdy AD, McCaffery JM, Shaw JM (2000). Dnm1p GTPase-mediated mitochondrial fission is a multi-step process requiring the novel integral membrane component Fis1p. J Cell Biol.

[87] Tieu Q, Nunnari J (2000). Mdv1p is a WD repeat protein that interacts with the dynamin-related GTPase, Dnm1p, to trigger mitochondrial division. J Cell Biol.

[88] Griffin EE, Graumann J, Chan DC (2005). The WD40 protein Caf4p is a component of the mitochondrial fission machinery and recruits Dnm1p to mitochondria. J Cell Biol.

[89] Osellame LD, Singh AP, Stroud DA (2016). Cooperative and independent roles of the Drp1 adaptors Mff, MiD49 and MiD51 in mitochondrial fission. J Cell Sci.

[90] Otera H, Wang C, Cleland MM (2010). Mff is an essential factor for mitochondrial recruitment of Drp1 during mitochondrial fission in mammalian cells. J Cell Biol.

[91] Palmer CS, Osellame LD, Laine D (2011). MiD49 and MiD51, new components of the mitochondrial fission machinery. EMBO Rep.

[92] Palmer CS, Elgass KD, Parton RG (2013). Adaptor proteins MiD49 and MiD51 can act independently of Mff and Fis1 in Drp1 recruitment and are specific for mitochondrial fission. J Biol Chem.

[93] Gandre-Babbe S, van der Bliek AM (2008). The novel tail-anchored membrane protein Mff controls mitochondrial and peroxisomal fission in mammalian cells. Mol Biol Cell.

[94] Macdonald PJ, Francy CA, Stepanyants N (2016). Distinct splice variants of dynamin-related protein 1 differentially utilize mitochondrial fission factor as an effector of cooperative GTPase activity. J Biol Chem.

[95] Zhao J, Liu T, Jin S (2011). Human MIEF1 recruits Drp1 to mitochondrial outer membranes and promotes mitochondrial fusion rather than fission. EMBO J.

[96] Losón OC, Song Z, Chen H, Chan DC (2013). Fis1, Mff, MiD49, and MiD51 mediate Drp1 recruitment in mitochondrial fission. Mol Biol Cell.

[97] Otera H, Miyata N, Kuge O, Mihara K (2016). Drp1-dependent mitochondrial fission via MiD49/51 is essential for apoptotic cristae remodeling. J Cell Biol.

[98] Nishida K, Takahara M, Miyagishima S (2003). Dynamic recruitment of dynamin for final mitochondrial severance in a primitive red alga. Proc Natl Acad Sci USA.

[99] Leger MM, Petrů M, Žárský V (2015). An ancestral bacterial division system is widespread in eukaryotic mitochondria. Proc Natl Acad Sci USA.

[100] Labrousse AM, Zappaterra MD, Rube DA, van der Bliek AM (1999). C. elegans dynamin-related protein DRP-1 controls severing of the mitochondrial outer membrane. Mol Cell.

[101] Fujioka H, Tandler B, Hoppel CL (2012). Mitochondrial division in rat cardiomyocytes: an electron microscope study. Anat Rec (Hoboken).

[102] Cho B, Cho HM, Jo Y (2017). Constriction of the mitochondrial inner compartment is a priming event for mitochondrial division. Nat Commun.

[103] Chakrabarti R, Ji W-K, Stan RV (2018). INF2-mediated actin polymerization at the ER stimulates mitochondrial calcium uptake, inner membrane constriction, and division. J Cell Biol.

[104] Kowaltowski AJ, Menezes-Filho SL, Assali EA (2019). Mitochondrial morphology regulates organellar Ca2+ uptake and changes cellular Ca2+ homeostasis. FASEB J.

[105] Szabadkai G, Simoni AM, Chami M (2004). Drp-1-dependent division of the mitochondrial network blocks intraorganellar Ca2+ waves and protects against Ca2+-mediated apoptosis. Mol Cell.

[106] Tondera D, Czauderna F, Paulick K (2005). The mitochondrial protein MTP18 contributes to mitochondrial fission in mammalian cells. J Cell Sci.

[107] Morita M, Prudent J, Basu K (2017). mTOR controls mitochondrial dynamics and cell survival via MTFP1. Mol Cell.

[108] Pagliuso A, Cossart P, Stavru F (2018). The ever-growing complexity of the mitochondrial fission machinery. Cell Mol Life Sci.

[109] Taguchi N, Ishihara N, Jofuku A (2007). Mitotic phosphorylation of dynamin-related GTPase Drp1 participates in mitochondrial fission. J Biol Chem.

[110] Cribbs JT, Strack S (2007). Reversible phosphorylation of Drp1 by cyclic AMP-dependent protein kinase and calcineurin regulates mitochondrial fission and cell death. EMBO Rep.

[111] Rambold AS, Kostelecky B, Elia N, Lippincott-Schwartz J (2011). Tubular network formation protects mitochondria from autophagosomal degradation during nutrient starvation. Proc Natl Acad Sci USA.

[112] Cereghetti GM, Stangherlin A, Martins de Brito O (2008). Dephosphorylation by calcineurin regulates translocation of Drp1 to mitochondria. Proc Natl Acad Sci USA.

[113] Wang H, Song P, Du L (2011). Parkin ubiquitinates Drp1 for proteasome-dependent degradation: implication of dysregulated mitochondrial dynamics in Parkinson disease. J Biol Chem.

[114] Karbowski M, Neutzner A, Youle RJ (2007). The mitochondrial E3 ubiquitin ligase MARCH5 is required for Drp1 dependent mitochondrial division. J Cell Biol.

[115] Park Y-Y, Lee S, Karbowski M (2010). Loss of MARCH5 mitochondrial E3 ubiquitin ligase induces cellular senescence through dynamin-related protein 1 and mitofusin 1. J Cell Sci.

[116] Park Y-Y, Nguyen OTK, Kang H, Cho H (2014). MARCH5-mediated quality control on acetylated Mfn1 facilitates mitochondrial homeostasis and cell survival. Cell Death Dis.

[117] Cherok E, Xu S, Li S (2017). Novel regulatory roles of Mff and Drp1 in E3 ubiquitin ligase MARCH5-dependent degradation of MiD49 and Mcl1 and control of mitochondrial dynamics. Mol Biol Cell.

[118] Toyama EQ, Herzig S, Courchet J (2016). Metabolism. AMP-activated protein kinase mediates mitochondrial fission in response to energy stress. Science.

[119] D’Amico D, Mottis A, Potenza F (2019). The RNA-binding protein PUM2 impairs mitochondrial dynamics and mitophagy during aging. Mol Cell.

[120] Soubannier V, McLelland G-L, Zunino R (2012). A vesicular transport pathway shuttles cargo from mitochondria to lysosomes. Curr Biol.

[121] Soubannier V, Rippstein P, Kaufman BA (2012). Reconstitution of mitochondria derived vesicle formation demonstrates selective enrichment of oxidized cargo. PLoS ONE.

[122] McLelland G-L, Soubannier V, Chen CX (2014). Parkin and PINK1 function in a vesicular trafficking pathway regulating mitochondrial quality control. EMBO J.

[123] McLelland G-L, Lee SA, McBride HM, Fon EA (2016). Syntaxin-17 delivers PINK1/parkin-dependent mitochondrial vesicles to the endolysosomal system. J Cell Biol.

[124] Sugiura A, Mattie S, Prudent J, McBride HM (2017). Newly born peroxisomes are a hybrid of mitochondrial and ER-derived pre-peroxisomes. Nature.

[125] Elmore SP, Qian T, Grissom SF, Lemasters JJ (2001). The mitochondrial permeability transition initiates autophagy in rat hepatocytes. FASEB J.

[126] Yamano K, Youle RJ (2013). PINK1 is degraded through the N-end rule pathway. Autophagy.

[127] Kondapalli C, Kazlauskaite A, Zhang N (2012). PINK1 is activated by mitochondrial membrane potential depolarization and stimulates Parkin E3 ligase activity by phosphorylating Serine 65. Open Biol.

[128] Shiba-Fukushima K, Arano T, Matsumoto G (2014). Phosphorylation of mitochondrial polyubiquitin by PINK1 promotes Parkin mitochondrial tethering. PLoS Genet.

[129] Lazarou M, Sliter DA, Kane LA (2015). The ubiquitin kinase PINK1 recruits autophagy receptors to induce mitophagy. Nature.

[130] Narendra D, Kane LA, Hauser DN (2010). p62/SQSTM1 is required for Parkin-induced mitochondrial clustering but not mitophagy; VDAC1 is dispensable for both. Autophagy.

[131] Nicot A-S, Lo Verso F, Ratti F (2014). Phosphorylation of NBR1 by GSK3 modulates protein aggregation. Autophagy.

[132] Yan C, Gong L, Chen L (2020). PHB2 (prohibitin 2) promotes PINK1-PRKN/Parkin-dependent mitophagy by the PARL-PGAM5-PINK1 axis. Autophagy.

[133] Yoshii SR, Kishi C, Ishihara N, Mizushima N (2011). Parkin mediates proteasome-dependent protein degradation and rupture of the outer mitochondrial membrane. J Biol Chem.

[134] Chan NC, Salazar AM, Pham AH (2011). Broad activation of the ubiquitin-proteasome system by Parkin is critical for mitophagy. Hum Mol Genet.

[135] Wei Y, Chiang W-C, Sumpter R (2017). Prohibitin 2 is an inner mitochondrial membrane mitophagy receptor. Cell.

[136] McWilliams TG, Prescott AR, Montava-Garriga L (2018). Basal mitophagy occurs independently of PINK1 in mouse tissues of high metabolic demand. Cell Metab.

[137] Lee JJ, Sanchez-Martinez A, Zarate AM (2018). Basal mitophagy is widespread in Drosophila but minimally affected by loss of Pink1 or parkin. J Cell Biol.

[138] Kitada T, Pisani A, Porter DR (2007). Impaired dopamine release and synaptic plasticity in the striatum of PINK1-deficient mice. Proc Natl Acad Sci USA.

[139] Goldberg MS, Fleming SM, Palacino JJ (2003). Parkin-deficient mice exhibit nigrostriatal deficits but not loss of dopaminergic neurons. J Biol Chem.

[140] Sandoval H, Thiagarajan P, Dasgupta SK (2008). Essential role for Nix in autophagic maturation of erythroid cells. Nature.

[141] Lee Y, Lee H-Y, Hanna RA, Gustafsson ÅB (2011). Mitochondrial autophagy by Bnip3 involves Drp1-mediated mitochondrial fission and recruitment of Parkin in cardiac myocytes. Am J Physiol Heart Circ Physiol.

[142] Chen G, Han Z, Feng D (2014). A regulatory signaling loop comprising the PGAM5 phosphatase and CK2 controls receptor-mediated mitophagy. Mol Cell.

[143] Liu L, Feng D, Chen G (2012). Mitochondrial outer-membrane protein FUNDC1 mediates hypoxia-induced mitophagy in mammalian cells. Nat Cell Biol.

[144] Wu W, Tian W, Hu Z (2014). ULK1 translocates to mitochondria and phosphorylates FUNDC1 to regulate mitophagy. EMBO Rep.

[145] Chen Z, Liu L, Cheng Q (2017). Mitochondrial E3 ligase MARCH5 regulates FUNDC1 to fine-tune hypoxic mitophagy. EMBO Rep.

[146] Murakawa T, Yamaguchi O, Hashimoto A (2015). Bcl-2-like protein 13 is a mammalian Atg32 homologue that mediates mitophagy and mitochondrial fragmentation. Nat Commun.

[147] Murakawa T, Okamoto K, Omiya S (2019). A mammalian mitophagy receptor, Bcl2-L-13, recruits the ULK1 complex to induce mitophagy. Cell Rep.

[148] Ljubicic V, Joseph A-M, Adhihetty PJ (2009). Molecular basis for an attenuated mitochondrial adaptive plasticity in aged skeletal muscle. Aging (Albany NY).

[149] Leduc-Gaudet J-P, Picard M, St-Jean Pelletier F (2015). Mitochondrial morphology is altered in atrophied skeletal muscle of aged mice. Oncotarget.

[150] Faitg J, Leduc-Gaudet J-P, Reynaud O (2019). Effects of aging and caloric restriction on fiber type composition, mitochondrial morphology and dynamics in rat oxidative and glycolytic muscles. Front Physiol.

[151] Iqbal S, Ostojic O, Singh K (2013). Expression of mitochondrial fission and fusion regulatory proteins in skeletal muscle during chronic use and disuse. Muscle Nerve.

[152] Romanello V, Guadagnin E, Gomes L (2010). Mitochondrial fission and remodelling contributes to muscle atrophy. EMBO J.

[153] Ogunbileje JO, Herndon DN, Murton AJ, Porter C (2018). The role of mitochondrial stress in muscle wasting following severe burn trauma. J Burn Care Res.

[154] Friedrich O, Reid MB, Van den Berghe G (2015). The sick and the weak: neuropathies/myopathies in the critically Ill. Physiol Rev.

[155] Gordaliza-Alaguero I, Cantó C, Zorzano A (2019). Metabolic implications of organelle-mitochondria communication. EMBO Rep.

[156] Barnes PJ (2017). Senescence in COPD and its comorbidities. Annu Rev Physiol.

[157] Barreto R, Mandili G, Witzmann FA (2016). Cancer and chemotherapy contribute to muscle loss by activating common signaling pathways. Front Physiol.

[158] van der Ende M, Grefte S, Plas R (2018). Mitochondrial dynamics in cancer-induced cachexia. Biochim Biophys Acta Rev Cancer.

[159] Brown JL, Rosa-Caldwell ME, Lee DE (2017). Mitochondrial degeneration precedes the development of muscle atrophy in progression of cancer cachexia in tumour-bearing mice. J Cachexia Sarcopenia Muscle.

[160] Sorrentino V, Menzies KJ, Auwerx J (2018). Repairing mitochondrial dysfunction in disease. Annu Rev Pharmacol Toxicol.

[161] Quirós PM, Español Y, Acín-Pérez R (2014). ATP-dependent Lon protease controls tumor bioenergetics by reprogramming mitochondrial activity. Cell Rep.

[162] Peter B, Waddington CL, Oláhová M (2018). Defective mitochondrial protease LonP1 can cause classical mitochondrial disease. Hum Mol Genet.

[163] Hannah-Shmouni F, MacNeil L, Brady L (2019). Expanding the clinical spectrum of LONP1-related mitochondrial cytopathy. Front Neurol.

[164] Nimmo GAM, Venkatesh S, Pandey AK (2019). Bi-allelic mutations of LONP1 encoding the mitochondrial LonP1 protease cause pyruvate dehydrogenase deficiency and profound neurodegeneration with progressive cerebellar atrophy. Hum Mol Genet.

[165] Hartmann B, Wai T, Hu H (2016). Homozygous YME1L1 mutation causes mitochondriopathy with optic atrophy and mitochondrial network fragmentation. Elife.

[166] Wai T, García-Prieto J, Baker MJ (2015). Imbalanced OPA1 processing and mitochondrial fragmentation cause heart failure in mice. Science.

[167] Quirós PM, Ramsay AJ, Sala D (2012). Loss of mitochondrial protease OMA1 alters processing of the GTPase OPA1 and causes obesity and defective thermogenesis in mice. EMBO J.

[168] Civitarese AE, MacLean PS, Carling S (2010). Regulation of skeletal muscle oxidative capacity and insulin signaling by the mitochondrial rhomboid protease PARL. Cell Metab.

[169] Cipolat S, Rudka T, Hartmann D (2006). Mitochondrial rhomboid PARL regulates cytochrome c release during apoptosis via OPA1-dependent cristae remodeling. Cell.

[170] Müller JMM, Deinhardt K, Rosewell I (2007). Targeted deletion of p97 (VCP/CDC48) in mouse results in early embryonic lethality. Biochem Biophys Res Commun.

[171] Watts GDJ, Wymer J, Kovach MJ (2004). Inclusion body myopathy associated with Paget disease of bone and frontotemporal dementia is caused by mutant valosin-containing protein. Nat Genet.

[172] Johnson JO, Mandrioli J, Benatar M (2010). Exome sequencing reveals VCP mutations as a cause of familial ALS. Neuron.

[173] Nalbandian A, Llewellyn KJ, Kitazawa M (2012). The homozygote VCPR155H/R155H mouse model exhibits accelerated human VCP-associated disease pathology. PLoS ONE.

[174] Bartolome F, Wu H-C, Burchell VS (2013). Pathogenic VCP mutations induce mitochondrial uncoupling and reduced ATP levels. Neuron.

[175] Kustermann M, Manta L, Paone C (2018). Loss of the novel Vcp (valosin containing protein) interactor Washc4 interferes with autophagy-mediated proteostasis in striated muscle and leads to myopathy in vivo. Autophagy.

[176] Viswanathan MC, Blice-Baum AC, Sang T-K, Cammarato A (2016). Cardiac-restricted expression of VCP/TER94 RNAi or disease alleles perturbs drosophila heart structure and impairs function. J Cardiovasc Dev Dis.

[177] Piccirillo R, Goldberg AL (2012). The p97/VCP ATPase is critical in muscle atrophy and the accelerated degradation of muscle proteins. EMBO J.

[178] Davies VJ, Hollins AJ, Piechota MJ (2007). Opa1 deficiency in a mouse model of autosomal dominant optic atrophy impairs mitochondrial morphology, optic nerve structure and visual function. Hum Mol Genet.

[179] Züchner S, Mersiyanova IV, Muglia M (2004). Mutations in the mitochondrial GTPase mitofusin 2 cause Charcot-Marie-Tooth neuropathy type 2A. Nat Genet.

[180] Alexander C, Votruba M, Pesch UE (2000). OPA1, encoding a dynamin-related GTPase, is mutated in autosomal dominant optic atrophy linked to chromosome 3q28. Nat Genet.

[181] Delettre C, Lenaers G, Griffoin JM (2000). Nuclear gene OPA1, encoding a mitochondrial dynamin-related protein, is mutated in dominant optic atrophy. Nat Genet.

[182] Schaaf CP, Blazo M, Lewis RA (2011). Early-onset severe neuromuscular phenotype associated with compound heterozygosity for OPA1 mutations. Mol Genet Metab.

[183] Amati-Bonneau P, Valentino ML, Reynier P (2008). OPA1 mutations induce mitochondrial DNA instability and optic atrophy “plus” phenotypes. Brain.

[184] Spiegel R, Saada A, Flannery PJ (2016). Fatal infantile mitochondrial encephalomyopathy, hypertrophic cardiomyopathy and optic atrophy associated with a homozygous OPA1 mutation. J Med Genet.

[185] Ibebunjo C, Chick JM, Kendall T (2013). Genomic and proteomic profiling reveals reduced mitochondrial function and disruption of the neuromuscular junction driving rat sarcopenia. Mol Cell Biol.

[186] Sebastián D, Sorianello E, Segalés J (2016). Mfn2 deficiency links age-related sarcopenia and impaired autophagy to activation of an adaptive mitophagy pathway. EMBO J.

[187] Tezze C, Romanello V, Desbats MA (2017). Age-associated loss of OPA1 in muscle impacts muscle mass, metabolic homeostasis, systemic inflammation, and epithelial senescence. Cell Metab.

[188] Bach D, Naon D, Pich S (2005). Expression of Mfn2, the Charcot-Marie-Tooth neuropathy type 2A gene, in human skeletal muscle: effects of type 2 diabetes, obesity, weight loss, and the regulatory role of tumor necrosis factor alpha and interleukin-6. Diabetes.

[189] Hernández-Alvarez MI, Thabit H, Burns N (2010). Subjects with early-onset type 2 diabetes show defective activation of the skeletal muscle PGC-1{alpha}/Mitofusin-2 regulatory pathway in response to physical activity. Diabetes Care.

[190] Chen H, Vermulst M, Wang YE (2010). Mitochondrial fusion is required for mtDNA stability in skeletal muscle and tolerance of mtDNA mutations. Cell.

[191] Ferreira JCB, Campos JC, Qvit N (2019). A selective inhibitor of mitofusin 1-βIIPKC association improves heart failure outcome in rats. Nat Commun.

[192] Rodríguez-Nuevo A, Díaz-Ramos A, Noguera E (2018). Mitochondrial DNA and TLR9 drive muscle inflammation upon Opa1 deficiency. EMBO J.

[193] Pereira RO, Tadinada SM, Zasadny FM (2017). OPA1 deficiency promotes secretion of FGF21 from muscle that prevents obesity and insulin resistance. EMBO J.

[194] Varanita T, Soriano ME, Romanello V (2015). The OPA1-dependent mitochondrial cristae remodeling pathway controls atrophic, apoptotic, and ischemic tissue damage. Cell Metab.

[195] Civiletto G, Varanita T, Cerutti R (2015). Opa1 overexpression ameliorates the phenotype of two mitochondrial disease mouse models. Cell Metab.

[196] Favaro G, Romanello V, Varanita T (2019). DRP1-mediated mitochondrial shape controls calcium homeostasis and muscle mass. Nat Commun.

[197] Romanello V, Scalabrin M, Albiero M (2019). Inhibition of the fission machinery mitigates OPA1 impairment in adult skeletal muscles. Cells.

[198] Ikeda Y, Shirakabe A, Maejima Y (2015). Endogenous Drp1 mediates mitochondrial autophagy and protects the heart against energy stress. Circ Res.

[199] Kageyama Y, Hoshijima M, Seo K, et al (2014) Parkin-independent mitophagy requires Drp1 and maintains the integrity of mammalian heart and brain. EMBO J 33:2798–2813. https://doi.org/10.15252/embj.201488658 10.15252/embj.201488658 PMC4282557 25349190

[200] Song M, Mihara K, Chen Y (2015). Mitochondrial fission and fusion factors reciprocally orchestrate mitophagic culling in mouse hearts and cultured fibroblasts. Cell Metab.

[201] Waterham HR, Koster J, van Roermund CWT (2007). A lethal defect of mitochondrial and peroxisomal fission. N Engl J Med.

[202] Yoon G, Malam Z, Paton T (2016). Lethal disorder of mitochondrial fission caused by mutations in DNM1L. J Pediatr.

[203] Vanstone JR, Smith AM, McBride S (2016). DNM1L-related mitochondrial fission defect presenting as refractory epilepsy. Eur J Hum Genet.

[204] Shamseldin HE, Alshammari M, Al-Sheddi T (2012). Genomic analysis of mitochondrial diseases in a consanguineous population reveals novel candidate disease genes. J Med Genet.

[205] Koch J, Feichtinger RG, Freisinger P (2016). Disturbed mitochondrial and peroxisomal dynamics due to loss of MFF causes Leigh-like encephalopathy, optic atrophy and peripheral neuropathy. J Med Genet.

[206] Bartsakoulia M, Pyle A, Troncoso-Chandía D (2018). A novel mechanism causing imbalance of mitochondrial fusion and fission in human myopathies. Hum Mol Genet.

[207] Chen H, Ren S, Clish C (2015). Titration of mitochondrial fusion rescues Mff-deficient cardiomyopathy. J Cell Biol.

[208] Ishihara N, Nomura M, Jofuku A (2009). Mitochondrial fission factor Drp1 is essential for embryonic development and synapse formation in mice. Nat Cell Biol.

[209] Wakabayashi J, Zhang Z, Wakabayashi N (2009). The dynamin-related GTPase Drp1 is required for embryonic and brain development in mice. J Cell Biol.

[210] Touvier T, De Palma C, Rigamonti E (2015). Muscle-specific Drp1 overexpression impairs skeletal muscle growth via translational attenuation. Cell Death Dis.

[211] Moore TM, Zhou Z, Cohn W (2019). The impact of exercise on mitochondrial dynamics and the role of Drp1 in exercise performance and training adaptations in skeletal muscle. Mol Metab.

[212] Oost LJ, Kustermann M, Armani A (2019). Fibroblast growth factor 21 controls mitophagy and muscle mass. J Cachexia Sarcopenia Muscle.

[213] Gutiérrez T, Simmen T (2018). Endoplasmic reticulum chaperones tweak the mitochondrial calcium rheostat to control metabolism and cell death. Cell Calcium.

[214] Rana A, Oliveira MP, Khamoui AV (2017). Promoting Drp1-mediated mitochondrial fission in midlife prolongs healthy lifespan of Drosophila melanogaster. Nat Commun.

[215] Murgia M, Toniolo L, Nagaraj N (2017). Single muscle fiber proteomics reveals fiber-type-specific features of human muscle aging. Cell Rep.

[216] Song J, Lei X, Jiao W (2018). Effect of Qiangji Jianli decoction on mitochondrial respiratory chain activity and expression of mitochondrial fusion and fission proteins in myasthenia gravis rats. Sci Rep.

[217] Song M, Franco A, Fleischer JA (2017). Abrogating mitochondrial dynamics in mouse hearts accelerates mitochondrial senescence. Cell Metab.

[218] Conte M, Ostan R, Fabbri C (2019). Human aging and longevity are characterized by high levels of mitokines. J Gerontol A Biol Sci Med Sci.

[219] Suomalainen A, Elo JM, Pietiläinen KH (2011). FGF-21 as a biomarker for muscle-manifesting mitochondrial respiratory chain deficiencies: a diagnostic study. Lancet Neurol.

[220] Kuma A, Hatano M, Matsui M (2004). The role of autophagy during the early neonatal starvation period. Nature.

[221] Komatsu M, Waguri S, Ueno T (2005). Impairment of starvation-induced and constitutive autophagy in Atg7-deficient mice. J Cell Biol.

[222] Lo Verso F, Carnio S, Vainshtein A, Sandri M (2014). Autophagy is not required to sustain exercise and PRKAA1/AMPK activity but is important to prevent mitochondrial damage during physical activity. Autophagy.

[223] Lira VA, Okutsu M, Zhang M (2013). Autophagy is required for exercise training-induced skeletal muscle adaptation and improvement of physical performance. FASEB J.

[224] Memme JM, Erlich AT, Phukan G, Hood DA (2019). Exercise and mitochondrial health. J Physiol (Lond).

[225] Greene JC, Whitworth AJ, Kuo I (2003). Mitochondrial pathology and apoptotic muscle degeneration in Drosophila parkin mutants. Proc Natl Acad Sci USA.

[226] Park J, Lee SB, Lee S (2006). Mitochondrial dysfunction in Drosophila PINK1 mutants is complemented by parkin. Nature.

[227] Clark IE, Dodson MW, Jiang C (2006). Drosophila pink1 is required for mitochondrial function and interacts genetically with parkin. Nature.

[228] Billia F, Hauck L, Konecny F (2011). PTEN-inducible kinase 1 (PINK1)/Park6 is indispensable for normal heart function. Proc Natl Acad Sci USA.

[229] Kravic B, Harbauer AB, Romanello V (2018). In mammalian skeletal muscle, phosphorylation of TOMM22 by protein kinase CSNK2/CK2 controls mitophagy. Autophagy.

[230] Raben N, Hill V, Shea L (2008). Suppression of autophagy in skeletal muscle uncovers the accumulation of ubiquitinated proteins and their potential role in muscle damage in Pompe disease. Hum Mol Genet.

[231] Masiero E, Agatea L, Mammucari C (2009). Autophagy is required to maintain muscle mass. Cell Metab.

[232] Ramesh I, Campos JC, Lee P (2019). Mitophagy protects against statin-mediated skeletal muscle toxicity. FASEB J.

[233] Romanello V, Sandri M (2015). Mitochondrial quality control and muscle mass maintenance. Front Physiol.

[234] Carnio S, LoVerso F, Baraibar MA (2014). Autophagy impairment in muscle induces neuromuscular junction degeneration and precocious aging. Cell Rep.

[235] Baraldo M, Geremia A, Pirazzini M (2020). Skeletal muscle mTORC1 regulates neuromuscular junction stability. J Cachexia Sarcopenia Muscle.

[236] Si H, Ma P, Liang Q (2019). Overexpression of pink1 or parkin in indirect flight muscles promotes mitochondrial proteostasis and extends lifespan in Drosophila melanogaster. PLoS ONE.

[237] Aparicio R, Rana A, Walker DW (2019). Upregulation of the autophagy adaptor p62/SQSTM1 prolongs health and lifespan in middle-aged drosophila. Cell Rep.

[238] Campos JC, Baehr LM, Gomes KMS (2018). Exercise prevents impaired autophagy and proteostasis in a model of neurogenic myopathy. Sci Rep.

[239] Campos JC, Queliconi BB, Bozi LHM (2017). Exercise reestablishes autophagic flux and mitochondrial quality control in heart failure. Autophagy.

[240] Cunha TF, Bechara LRG, Bacurau AVN (2017). Exercise training decreases NADPH oxidase activity and restores skeletal muscle mass in heart failure rats. J Appl Physiol.

[241] Castets P, Frank S, Sinnreich M, Rüegg MA (2016). “Get the balance right”: pathological significance of autophagy perturbation in neuromuscular disorders. J Neuromuscul Dis.

[242] Nemazanyy I, Blaauw B, Paolini C (2013). Defects of Vps15 in skeletal muscles lead to autophagic vacuolar myopathy and lysosomal disease. EMBO Mol Med.

[243] Grumati P, Coletto L, Sabatelli P (2010). Autophagy is defective in collagen VI muscular dystrophies, and its reactivation rescues myofiber degeneration. Nat Med.

[244] Ramos FJ, Chen SC, Garelick MG (2012). Rapamycin reverses elevated mTORC1 signaling in lamin A/C-deficient mice, rescues cardiac and skeletal muscle function, and extends survival. Sci Transl Med.

[245] De Palma C, Morisi F, Cheli S (2012). Autophagy as a new therapeutic target in Duchenne muscular dystrophy. Cell Death Dis.

[246] Pauly M, Daussin F, Burelle Y (2012). AMPK activation stimulates autophagy and ameliorates muscular dystrophy in the mdx mouse diaphragm. Am J Pathol.

